# Physiologically based Kinetic Modeling-Facilitated Quantitative *In Vitro* to *In Vivo* Extrapolation to Predict the Effects of Aloe-Emodin in Rats and Humans

**DOI:** 10.1021/acs.jafc.4c00969

**Published:** 2024-07-09

**Authors:** Qiuhui Ren, Jiaqi Chen, Sebastiaan Wesseling, Hans Bouwmeester, Ivonne M. C. M. Rietjens

**Affiliations:** Division of Toxicology, Wageningen University and Research, Stippeneng 4, Wageningen 6708 WE, The Netherlands

**Keywords:** physiologically based kinetic (PBK) modeling, quantitative *in vitro* to *in vivo* extrapolation (QIVIVE), hepatotoxicity, nephrotoxicity, reactive oxidative stress (ROS), nuclear factor erythroid 2-related factor 2 (Nrf2)

## Abstract

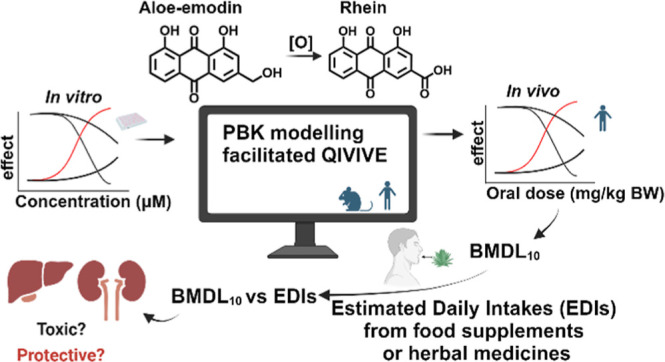

Aloe-emodin, a natural hydroxyanthraquinone, exerts both adverse and protective effects. This study aimed at investigating these potential effects of aloe-emodin in humans upon the use of food supplements and herbal medicines using a physiologically based kinetic (PBK) modeling-facilitated quantitative *in vitro* to *in vivo* extrapolation (QIVIVE) approach. For this, PBK models in rats and humans were established for aloe-emodin including its active metabolite rhein and used to convert *in vitro* data on hepatotoxicity, nephrotoxicity, reactive oxidative species (ROS) generation, and Nrf2 induction to corresponding *in vivo* dose–response curves, from which points of departure (PODs) were derived by BMD analysis. The derived PODs were subsequently compared to the estimated daily intakes (EDIs) resulting from the use of food supplements or herbal medicines. It is concluded that the dose levels of aloe-emodin from food supplements or herbal medicines are unlikely to induce toxicity, ROS generation, or Nrf2 activation in liver and kidney.

## 1 Introduction

Aloe-emodin, also named 1,8-dihydroxy-3-hydroxymethyl-anthraquinone, is a hydroxyanthraquinone naturally occurring in various plant species, such as *Aloe vera*, *Rheum palmatum* L., *Polygonum multiflorum* Thunb, and *Cassia occidentlis*, which are not only traditionally used as ingredients in Chinese herbal medicines but also globally recognized and widely used as food or food supplements.^1−4^ Many *in vitro* and *in vivo* (rat and mouse) studies have reported that aloe-emodin has a range of biological activities and thus has diverse therapeutic potential, resulting in claimed antiviral, anti-inflammatory, anticancer, antibacterial, and immunomodulatory effects.^5^ Activation of the Nuclear factor E2-related factor 2 (Nrf2) signaling pathway is one of the proposed key modes of action underlying the beneficial effects of aloe-emodin.^6^ Nrf2 can be activated by aloe-emodin resulting in the release of Nrf2 from Kelch-like ECH-associated protein 1 (Keap1), its subsequent translocation into the nucleus followed by induction of downstream cytoprotective gene expression, such as heme oxygenase 1 (HO-1) and NAD(P)H: quinone oxidoreductase 1 (NQO1).^6^ However, hepatotoxicity and nephrotoxicity induced by aloe-emodin have also been observed in both *in vitro* (Figures S1 and S2) and *in vivo* (mouse) studies.^7^ In these studies, it was shown that aloe-emodin induced apoptosis in HepaRG cells in a concentration- and time- dependent manner by generating reactive oxygen species (ROS) and depolarizing the mitochondrial membrane potential.^5^

**Figure 1:**
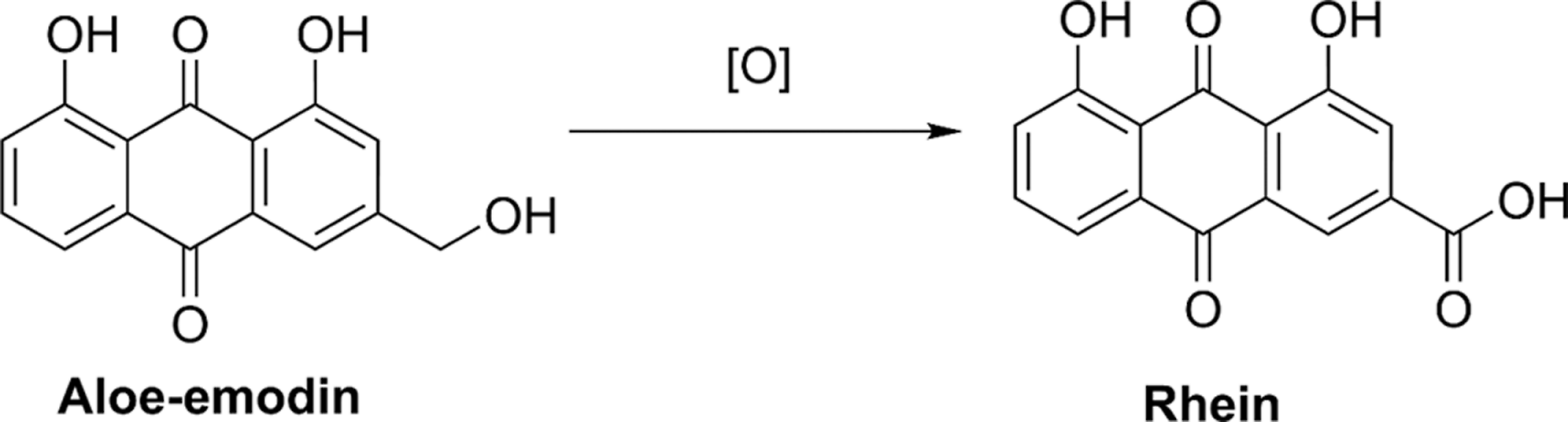
Metabolic conversion of aloe-emodin to rhein.^8,9^

The aim of the present study was to investigate whether current dose levels of aloe-emodin exposure resulting from food supplements or herbal medicines would result in these different effects in humans using physiologically based kinetic (PBK) modeling-facilitated quantitative *in vitro* to *in vivo* extrapolation (QIVIVE) as a new approach methodology. Previous *in vivo* studies have reported that aloe-emodin is metabolized to rhein^8,9^ (Figure 1), which was also reported to be able to induce hepatotoxicity, nephrotoxicity, ROS generation, and/or Nrf2 activation.^10^ Therefore, the activity of the metabolite rhein and its contribution to hepatotoxicity, nephrotoxicity, ROS generation, and Nrf2 activation following *in vivo* aloe-emodin administration were also taken into account by using aloe-emodin equivalents obtained from relative potency factors (RPFs). Additionally, aloe-emodin glucuronides were not considered since glucuronidation generally nullifies biological effects or activities.^11^ For either aloe-emodin or rhein, no compound accumulation was expected, given that the available literature studies showed full clearance of aloe-emodin within 24 h, and rhein was reported to show rapid distribution and did not accumulate in the organs.^8,12^

In the present study, *in vitro* and *in silico* methods were used as new approach methodologies (NAMs) to quantify *in vivo* dose–response curves for aloe-emodin induced hepatotoxicity, nephrotoxicity, ROS generation, or Nrf2 activation in rats and humans, without generating new animal data. To this end, PBK models for aloe-emodin and the active metabolite rhein for rats and humans were developed. The kinetic parameters of aloe-emodin and rhein were obtained from the literature or derived from *in silico* predictions as well as *in vitro* incubations. The PBK models were used to translate *in vitro* concentration–response curves for hepatotoxicity, nephrotoxicity, ROS generation, and Nrf2 activation to corresponding *in vivo* dose–response curves, enabling prediction of *in vivo* dose-dependent hepatotoxicity, nephrotoxicity, ROS generation, and Nrf2 activation. Application of such a PBK modeling-facilitated QIVIVE approach contributes to the development of NAMs for the safety assessment of chemicals within the framework of replacing, reducing, and refining (3Rs) the use of animal experiments and enabling predictions for humans without the need for human intervention studies.

## 2 Materials and Methods

### 2.1 General Outline of PBK Modeling-based QIVIVE Approach and BMD Analysis

To assess the potential protective and toxic effects of aloe-emodin in humans from dietary and medical intake, PBK modeling-facilitated QIVIVE and BMD analysis were performed in the following steps: (1) rat and human PBK models were developed for aloe-emodin and its bioactive metabolite (rhein); (2) the performance of the rat model was evaluated by comparing the model predictions to available *in vivo* toxicokinetic data in rats;^8,13^ and the human model was assumed to perform well, as relevant data were unavailable for its evaluation; (3) *in vitro* concentration-based hepatotoxicity, nephrotoxicity, ROS generation, and Nrf2 activation induced by aloe-emodin and rhein were either obtained from reported human cell data shown in Figures S1–S3 or determined with *in vitro* human cell assays in the present study (Sections 2.5 and 2.6); (4) with PBK model-based QIVIVE, the *in vitro* concentration–response data sets were extrapolated to corresponding *in vivo* dose–response curves considering rhein’s contribution by using aloe-emodin equivalents obtained from RPFs (Section 2.7); and (5) BMD analysis was performed on the predicted dose–response curves to derive the corresponding dose levels at which no induction of the target effects (hepatotoxicity, nephrotoxicity, ROS generation, and Nrf2 activation) takes place, and the predicted dose levels were finally compared to the estimated daily intakes (EDIs) of aloe-emodin from food supplements and herbal medicines. EDI values were calculated according to the contents of aloe-emodin in these food supplements or herbal medicines and the recommended daily usage of food supplements by suppliers as well as the recommended usage of herbal medicines based on the Chinese Pharmacopoeia 2020 edition (Tables S3 and S4).

### 2.2 *In Vitro* Incubations to Derive the Kinetic Parameters for the PBK Models

#### 2.2.1 Biotransformation of Aloe-Emodin to Rhein by Rat and Human Liver Microsomes

The biotransformation of aloe-emodin to rhein was determined using *in vitro* human and rat liver microsomal incubations. Preliminary experiments were performed to optimize the incubation time and concentration of microsomal protein, resulting in conditions in which metabolism was linear with respect to time and microsomal protein quantity (data not shown). The final incubations contained 50 mM Tris-HCl (pH 7.4), 5 mM MgCl_2_, 1 mM NADPH, and aloe-emodin at various concentrations {0 [1% (v/v) DMSO as solvent control], 0.5, 1, 2, 5, 10, 20, 50, and 100 μM}, added from 100 times concentrated stock solutions in DMSO. After 1 min preincubation in a water bath at 37 °C, 0.5 μL of rat liver microsomes (final concentration 0.05 mg microsomal protein/mL) or 1 μL of human liver microsomes (final concentration 0.1 mg microsomal protein/mL) were added to initiate the reaction. The total volume of the incubations was 200 μL. The same incubations were performed where NADPH was replaced with buffer to serve as controls. The reaction was terminated after 5 min by adding 100 μL of ice-cold ACN, and samples were kept on ice for 15 min. Subsequently, the samples were subjected to centrifugation at 16,000*g* for 5 min at 4 °C. The supernatants were transferred to vials and analyzed using LC-MS/MS to quantify the formation of the metabolite, rhein (Supporting Information, Supporting Information Materials and Methods). Incubations were performed in triplicate.

#### 2.2.2 Glucuronidation of Aloe-Emodin by Rat and Human Liver S9 Fractions

In addition to its conversion to rhein, aloe-emodin was also reported to be metabolized to glucuronide conjugates.^8^ To quantify the kinetic parameters required to include this clearance in the PBK model, *in vitro* incubations with pooled liver S9 fractions from rats and humans were performed. Before kinetic studies, incubation time and liver S9 concentration were optimized to determine the conditions for linearity in time and with the amount of S9 protein added (data not shown). The final incubations contained 50 mM Tris-HCl (pH 7.4), 10 mM MgCl_2_, 10 mM UDPGA, 0.025 mg/mL of alamethicin, and increasing concentrations of aloe-emodin {0 [1% (v/v) DMSO as solvent control], 0.5, 1, 2, 5, 10, 20, 50, 75, and 100 μM}, which were added from 100 times the concentrated stock solutions in DMSO. After preincubation in a water bath at 37 °C for 1 min, 2.5 μL of rat or human S9 fraction (final concentration 0.05 mg protein/mL) was added to initiate the reaction. Incubations without aloe-emodin or without UDPGA served as negative and solvent controls, respectively. After incubating 30 min for rat samples or 60 min for human samples in a water bath at 37 °C, 25 μL of ice-cold ACN was added to terminate the reaction. Subsequently, samples were centrifuged at 21,500*g* for 5 min at 4 °C to precipitate proteins. Supernatants were used for quantification of the aloe-emodin glucuronides by UPLC analysis (Supporting Information Materials and Methods).

The formation of aloe-emodin glucuronides was confirmed using β-glucuronidase-mediated hydrolysis, resulting in the disappearance of the peaks of aloe-emodin glucuronides with a corresponding increase of the aloe-emodin peak. To this end, 50 μL of nonterminated incubation samples prepared as described above were added to 50 μL 50 mM Tris-HCl (pH 7.4) containing 200 units/mL β-glucuronidase. The mixture was incubated for 2 h in a water bath at 37 °C, and subsequently, 25 μL of ice-cold ACN was added to terminate the reaction. The mixture was centrifuged at 21,500*g* for 5 min at 4 °C, and the supernatants were collected for quantification of the aloe-emodin glucuronides and aloe-emodin by UPLC-PDA analysis (Supporting Information Materials and Methods).

#### 2.2.3 Hepatic Clearance of Rhein by Primary Rat Hepatocytes

The intrinsic clearance (CL_int_) of rhein derived from hepatocyte incubations was available for humans.^14^ For rats, the kinetic parameters for hepatic clearance of rhein were quantified by using *in vitro* rat hepatocyte incubations. To this end, pooled primary hepatocytes were thawed and diluted to a target density of 1 × 10^6^ cells/mL based on the method described in a previous study.^15^ The exposure medium was composed of an incubation medium containing 2 μM rhein added from a 2 mM stock in DMSO (final DMSO concentration 0.1% v/v). Before starting the incubation, the exposure medium was preincubated for 5 to 10 min at 37 °C. The incubation was started by adding 100 μL of primary hepatocytes into 100 μL of preincubated exposure medium, resulting in a final concentration of 0.5 × 10^6^ cells/mL and 1 μM rhein (final DMSO concentration 0.05% v/v). The samples were incubated on a shaker (Titramax 1000, Heidolph, Germany) at 150 rpm in a humidified incubator containing 5% CO_2_ at 37 °C. The time points for the incubation were 0, 2, 5, 10, 15, 20, 30, 40, 60, 90, and 120 min. A control was included for each incubation time point, consisting of an incubation medium without primary hepatocytes. To terminate the reactions at the indicated time points, 100 μL of aliquot of the incubation was transferred to an Eppendorf tube containing 50 μL of ice-cold ACN, and the samples were kept on ice for 15 min. Subsequently, the samples were centrifuged at 16,000*g* for 5 min at 4 °C. The supernatants were collected, and the remaining rhein was quantified *via* LC-MS/MS analysis (Supporting Information Materials and Methods).

### 2.3 Definition of the PBK Model for Aloe-Emodin Including a Submodel for Rhein

Rat and human PBK models were coded in Berkeley Madonna (version 10.5.1, UC Berkeley, CA, USA), applying Rosenbrock’s algorithms for solving stiff systems. Model codes in rats and humans are provided in the Supporting Information. The physiological parameters for the rat and human PBK models are summarized in Table S1.

**Figure 2:**
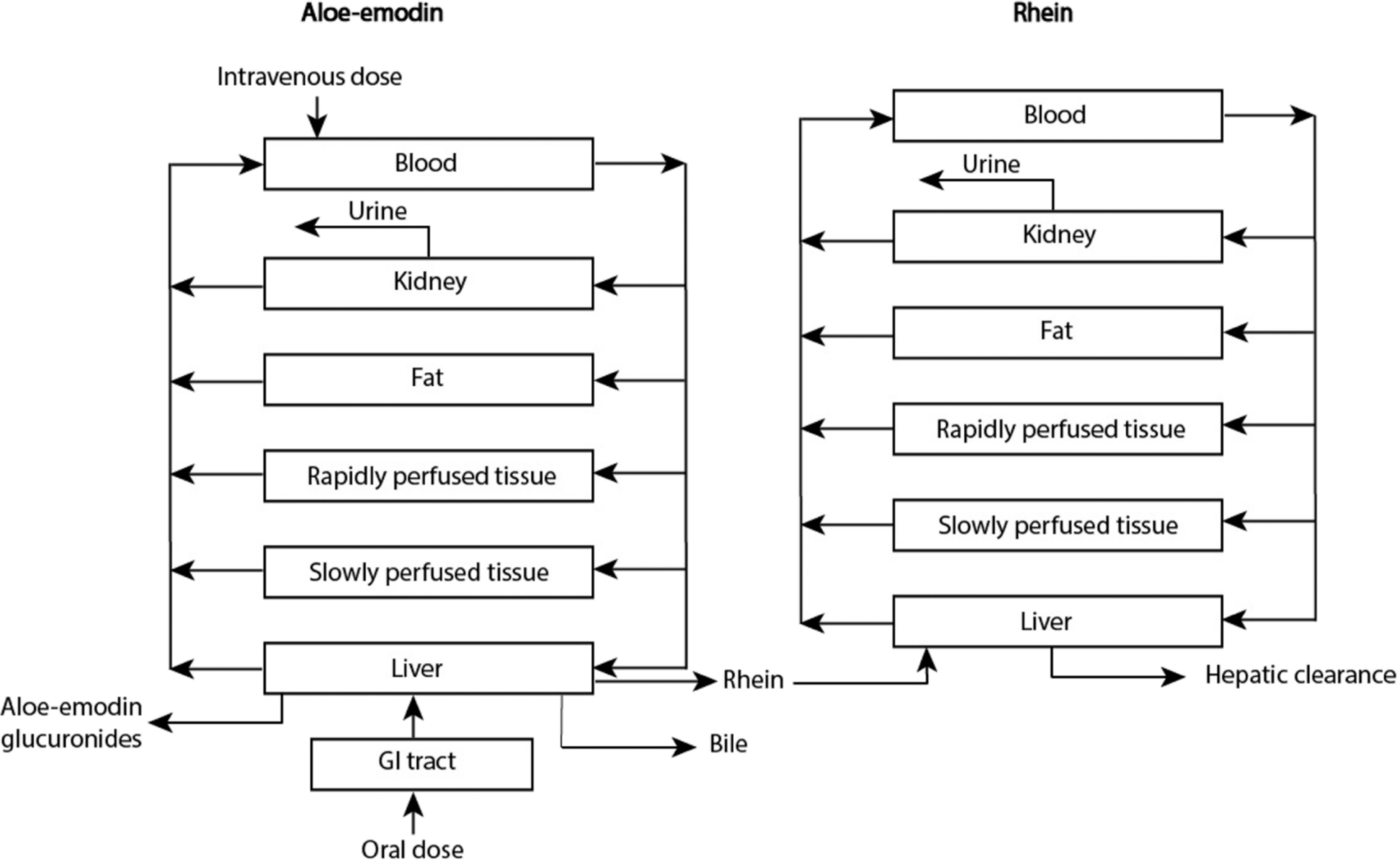
Schematic overview of the PBK model for aloe-emodin with a submodel for rhein.

The established PBK models in this study described the absorption, distribution, metabolism, and excretion of aloe-emodin and its metabolite rhein in rats or humans. Figure 2 shows the schematic overview of the PBK model for aloe-emodin including a submodel for its metabolite rhein. The submodel for rhein was developed to predict to what extent its internal concentrations formed upon conversion of aloe-emodin would contribute to hepatotoxicity, nephrotoxicity, ROS generation, and/or Nrf2 activation in the liver and kidney. The PBK model consisted of different compartments, including GI tract, liver, slowly perfused tissues (skin, muscle, and bone), rapidly perfused tissues (brain, heart, and lung), fat, kidney, and blood.

A PBK model for single oral dose administration was developed given that this is a major exposure route for aloe-emodin from dietary and medicinal intake, and the available data sets for model performance evaluation were obtained *in vivo* upon single exposure. The intestinal absorption of aloe-emodin was reported to be passive diffusion.^16^ The oral absorption rate constant (*ka*) and fraction of dose absorbed (*Fa*) were then predicted by quantitative structure–activity relationship (QSAR) tools. Briefly, the *in vitro* apparent permeability coefficient (Log *P*_app_) in Caco2 cell model was predicted to be (−0.233 × 10^–6^ cm/s) by pkCSM.^17^ The *ka* and *Fa* in rats and humans were calculated by eqs 1–4
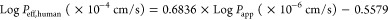
1
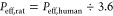
2
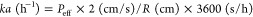
3
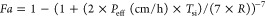
4where eq 1 indicates *in vitro* to *in vivo* scaling from Log *P*_app_ for the Caco2 model to the human effective permeability (Log *P*_eff,human_) for passive diffusion.^18^Equation 2 was used to calculate rat *P*_eff_ by dividing human *P*_eff_ by the interspecies scaling factor.^19^ In eqs 3 and 4,^20^*R* represents the radius of the small intestine, which is 0.18 and 1 cm for rats and humans, respectively,^19,21^ and *T*_si_ (the small intestinal transit time) is 1.47 h for rats and 3.32 h for humans.^19,21^ Thus, the calculated *ka* amounts to 0.21 and 0.14 h^–1^ in rats and humans, respectively, while the predicted *Fa* amounts to 0.26 for rats and 0.36 for humans. Besides, an intravenous (i.v.) route was also added into the model to enable the evaluation of model performance by comparison to available kinetic data sets in rats upon I.V. dosing.

The distribution of aloe-emodin and rhein across tissues was described with tissue/blood partition coefficients. The tissue/plasma partition coefficients were first predicted using the Rodger and Rowland method facilitated by an online QIVIVE tools (version 2.0)^22,23^ with acid–base properties (p*K*_a_) and lipophilicity (Log *P*) as inputs (Table S2). To obtain the tissue/blood partition coefficients, the tissue/plasma partition coefficients were then divided by the blood/plasma ratio (BPR) to correct for the difference in compound distribution between blood and plasma. The BPR of aloe-emodin (BPR_aloe-emodin_), as an acidic compound, was assumed to be 0.55 (1-hematocrit),^24^ and the BPR for rhein (BPR_rhein_) was reported to be 0.95 and 0.96 for rats and humans, respectively.^25^

Hepatic clearance of aloe-emodin and rhein was assumed to take place only in the liver compartment. Kinetic parameters, such as apparent maximum reaction rate, Michaelis–Menten constant, and *in vitro* clearance rate (*V*_max_, *K*_m_, and CL_int, *in vitro*_), were obtained from *in vitro* incubations (Section 2.2). The *K*_m_ determined *in vitro* was assumed to be equal to the *K*_m_*in vivo*. The *in vitro V*_max_ for the conversion of aloe-emodin to rhein was scaled to the liver using microsomal protein contents of 46 and 40 mg microsomal protein/g liver for rats and humans, respectively.^22,26^ The *in vitro V*_max_ for glucuronidation of aloe-emodin was scaled to the liver using a scaling factor of 165 mg S9 protein/g liver for rats^27^ and 120.7 mg S9 protein/g liver for humans (comprising 40 mg microsomal protein and 80.7 mg of cytosolic protein).^28^ For rhein, the hepatic metabolic clearance of rhein (CL_int_) in rats was scaled to the liver with a scaling factor of 135,000 million cells/kg liver,^29^ and the reported CL_int, *in vitro*_ of rhein is 0 for humans.^14^

Biliary and renal elimination were included in the models as excretion routes for aloe-emodin, while for rhein, only renal clearance was taken into account, as biliary excretion is not dominant.^43^ The biliary excretion of aloe-emodin was described by a bile excretion constant (kb) which was assumed to be 1.^30^ Glomerular filtration was assumed to be the main route for urinary excretion,^15^ and the glomerular filtration rates in rats and humans used in the models were 5.2 and 1.8 mL/min/kg body weight (BW), respectively.^31^

### 2.4 Evaluation of the PBK Model

To assess the performance of the rat PBK model, the predicted blood concentrations of aloe-emodin and rhein were compared to the corresponding blood concentrations retrieved from *in vivo* studies employing single intravenous or oral dose administration of aloe-emodin. Given the lack of available human *in vivo* data, the predictions by the human model could not be evaluated separately but were assumed to be validated by adequate performance of the comparable rat PBK model.^15^ The *in vivo* data on time-dependent blood concentrations of aloe-emodin or rhein in rats were extracted from graphs presented in the respective articles using TechDig 2.0 and processed in Prism GraphPad (version 5.04, San Diego, CA, USA).

A local sensitivity analysis was performed on all model input parameters to identify influential parameters in the PBK models (rats and humans) on the predicted maximum blood concentration (*C*_max_) of aloe-emodin and rhein. The description of the method and results is provided in Supporting Information Materials and Methods and Figure S8.

### 2.5 Cytotoxicity to Human Liver and Human Kidney Cells

The human hepatoma HepG2 cells and the human kidney HK-2 cells were provided by an American type of culture collection (Manassas, Virginia). Cells were cultured in DMEM/F12 containing 10% (v/v) FBS and penicillin/streptomycin (P/S, final concentrations 10 U/mL and 10 μg/mL, respectively) and incubated at 37 °C with 5% CO_2_. Cells were subcultured every 3 or 4 days.

To quantify the cytotoxicity of aloe-emodin and rhein, HepG2 cells were seeded at a density of 2 × 10^4^ cells/well, while HK-2 cells were seeded at a density of 4.5 × 10^3^ cells/well. Both were seeded in 96-well plates and incubated at 37 °C with 5% (v/v) CO_2_ overnight. Then, the cells were exposed to aloe-emodin or rhein for 24 h at different concentrations {0 [1% (v/v) DMSO as solvent control], 0.6, 2, 6, 10, 20, 60, and 100 μM}, added from 100-fold concentrated stock solutions in DMSO resulting in a final concentration of DMSO of 1% (v/v). Due to the limited solubility of both aloe-emodin and rhein, concentrations exceeding 100 μM could not be tested. After exposure, 5% (v/v) WST-1 regent was added in each well, and the plates were incubated for 1 h at 37 °C with 5% (v/v) CO_2_. Subsequently, the absorbance at 440 and 620 nm was measured by a SpectraMax M2 (Molecular Devices, USA). The data were expressed as cell viability (%) compared to solvent control which set as 100% cell viability.

### 2.6 ROS Generation Using a Human Cell-based Bioassay

The Nrf2 CALUX cells (BDS, Amsterdam, The Netherlands), which are modified human osteoblastic osteosarcoma U2OS cells, were also used for the Nrf2 CALUX assay that quantified Nrf2 activation by aloe-emodin and rhein in our previous study.^6^ The cells were cultured in DMEM/F12 medium supplemented with 7.5% (v/v) FBS, 1% (v/v) NEAA and P/S (10 U/mL and 10 μg/mL, respectively) and maintained in a humidified atmosphere with 5% (v/v) CO_2._ The cells were subcultured every 3 or 4 days. Additionally, the antibiotic G418, also known as Geneticin, was added at a final concentration of 0.2 mg/mL once a week to maintain a clean culture of transfected CALUX cells which are G418 resistant,^32^

To quantify ROS generation of aloe-emodin and rhein in Nrf2 CALUX cells, the DCF-DA assay was performed essentially as previously described.^33^ The cells were seeded in the 60 inner wells of black 96-well plates at a density of 3.0 × 10^4^ cells per well in 100 μL of growth medium. In outer wells, 200 μL of PBS was added. Plates were incubated at 37 °C with 5% v/v CO_2_ in a humidified atmosphere for 24 h in order to form confluent cell layers. The growth medium was then removed, and the cells were washed with 100 μL prewarmed PBS (37 °C) per well. Subsequently, a supplemented buffer [PBS with 0.4% (v/v) FBS] containing 25 μM H_2_DCF-DA was introduced into each well. The cells were then incubated for 60 min at 37 °C in a humidified atmosphere with 5% (v/v) CO_2_. After removing the H_2_DCF-DA containing buffer, the cells were subjected to aloe-emodin or rhein at 0 [1% (v/v) DMSO as solvent control], 2, 6, 10, 20, 60, and 100 μM in 100 μL of assay medium (DMEM/F12 without phenol red) per well for 4 h. The previous study in which Cytotox CALUX cells were exposed to aloe-emodin and rhein already showed that the exposure concentrations used do not cause cytotoxicity in the CALUX cells.^6^ Following this incubation, the fluorescence intensities were assessed at λ_exc_ 485 and λ_emm_ 535 nm using a SpectraMax iD3 instrument (Molecular Devices, San Jose, USA).

### 2.7 Determination of Aloe-Emodin Equivalents by Relative Potency Factor

Based on *in vitro* data, the hepatotoxicity, nephrotoxicity, ROS generation, and Nrf2 activation of rhein were expressed in aloe-emodin equivalents by using RPF values. The RPF of aloe-emodin was set as 1. The RPF of rhein (RPF_rhein_) for the different end points was calculated by the following equation (eq 5) using the BMCL_10_ values of the respective *in vitro* bioassays
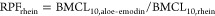
5where BMCL_10_ is identified as the lower confidence bound of benchmark concentration producing an extra 10% response above background compared to the control, and it was obtained using the European Food Safety Authority (EFSA) online BMD analysis tool (https://r4eu.efsa.europa.eu/app/bmd) integrated with the R package PROAST version 70.0.

### 2.8 Model Application for QIVIVE and the Point of Departure Derivation

*In vitro* concentration–response data for four end points (hepatotoxicity, nephrotoxicity, ROS generation, and Nrf2 activation) were derived from experiments conducted in the present study, as described in Sections 2.5 and 2.6, Additionally, to supplement our findings, literature-reported data on the toxicity of aloe-emodin in other *in vitro* cell models were collected (Figures S1–S3). This included data from literature on *in vitro* hepatotoxicity and nephrotoxicity quantified with the MTT or CKK-8 assay under increasing concentrations of aloe-emodin in human liver HepG2, HepaRG, and HL7702 cells, or kidney HK-2 cells (Figures S1–S3). The data for aloe-emodin- and rhein-mediated Nrf2 activation were taken from our previous study using the Nrf2 CALUX reporter gene assay.^6^ For all data, the effective *in vitro* concentrations of unbound aloe-emodin expressed as aloe-emodin equivalents were set equal to the unbound *in vivo* maximum venous blood concentration in the liver or kidney, expressed in aloe-emodin equivalents, following the equations (eqs 6 and 7)
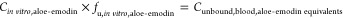
6
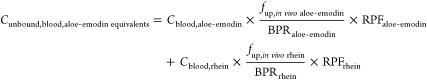
7in which *C*_*in vitro*, aloe-emodin_ and *f*_u, *in vitro*, aloe-emodin_ are the *in vitro* concentration and the unbound fraction of aloe-emodin in the medium of the *in vitro* assay, respectively. *C*_blood, aloe-emodin_ and *C*_blood, rhein_ are the total blood concentrations of aloe-emodin and rhein, respectively. BPR_aloe-emodin_ and BPR_rhein_ are the respective BPRs of aloe-emodin and rhein (0.55 and 0.96, respectively). *f*_up, *in vivo*, aloe-emodin_ and *f*_up, *in vivo*, rhein_ are the *in vivo* plasma unbound fractions of aloe-emodin and rhein, which are 0.092 (predicted by QIVIVE toolbox)^22^ and 0.91,^25^ respectively. RPF_aloe-emodin_ is the RPF of aloe-emodin defined as 1.0, and RPF_rhein_ is the RPF of rhein relative to aloe-emodin for the respective end point calculated from results of the relevant *in vitro* assay, as indicated in Section 2.7. The *f*_u, *in vitro*, aloe-emodin_ values were predicted to be 0.79 and 0.65, respectively, following eq 8(34)
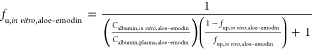
8in which *C*_albumin, *in vitro*, aloe-emodin_ and *C*_albumin, plasma, aloe-emodin_ are the concentrations of albumin used in *in vitro* assay and in human plasma (42.5 g/L),^35^ respectively. In exposure medium of *in vitro* assay, 5% (v/v) FBS and 10% (v/v) can result in 1.15 and 2.3 g/L fetal bovine albumin,^36^ respectively. Thus, the *f*_u, *in vitro*, aloe-emodin_ values for the reported *in vitro* data sets for Nrf2 activation with exposure medium containing 5% (v/v) FBS and for hepatoxicity and nephrotoxicity with exposure medium containing 10% (v/v) FBS, as reported in the literature.

Furthermore, to determine the point of departure (PODs) from PBK modeling predicted *in vivo* curves, the aforementioned BMD analysis tool was used to obtain BMDL_10_ (the lower confidence limit of the concentration giving 10% response above background) and BMDU_10_ (the upper confidence limit of the concentration giving 10% response above background), which represents the lower and upper confidence bounds of the benchmark dose, respectively, that produced an extra 10% response above background compared to the control because the end points of interest were assumed to be thresholded and thus dependent on *C*_max_ as the relevant parameter for the reverse dosimetry.

## 3 Results

The aim of the present study was to investigate whether daily intakes of aloe-emodin *via* use of food supplements or herbal medicines would cause adverse or beneficial health effects in humans using a PBK modeling-facilitated QIVIVE approach. To define the kinetic PBK model parameters, the kinetic parameters for biotransformation of aloe-emodin to rhein, glucuronidation of aloe-emodin, and hepatic clearance of rhein in the liver were determined and subsequently incorporated in the PBK models for rats and humans. After validation of the PBK models by comparing to available *in vivo* data, the QIVIVE approach was used to translate a series of *in vitro* concentration–response curves to *in vivo* dose–response curves, from which PODs were derived by BMD analysis. The derived PODs were subsequently compared to EDIs resulting from the use of food supplements or herbal medicines, elucidating if daily intakes of aloe-emodin by food supplements or herbal medicines would be likely to cause various health effects in humans.

### 3.1 Kinetic Parameters for Biotransformation of Aloe-Emodin

**Figure 3:**
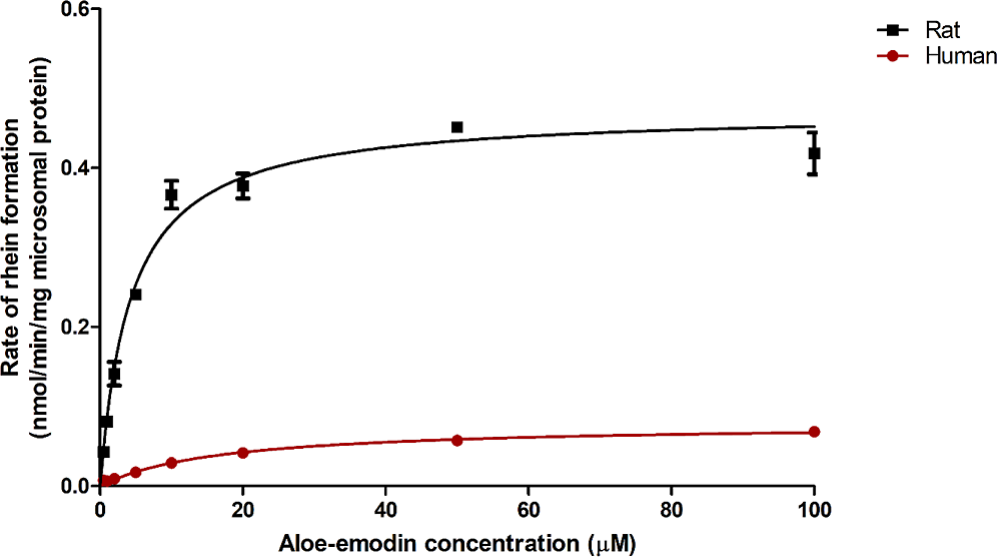
Aloe-emodin concentration-dependent formation of rhein in incubations with rat (black square) and human (red triangle) liver microsomes. Data points represent means ± SEM of three experiments for each conversion.

Figure 3 shows the concentration-dependent formation of rhein in incubations of aloe-emodin with pooled rat or human liver microsomes. Interspecies differences in rhein formation were noted, where rats could convert aloe-emodin to rhein faster than humans. The kinetic parameters derived from these curves including unscaled and scaled *V*_max_ values, *K*_m_ as well as unscaled and scaled catalytic efficiency (CE, calculated as *V*_max_/*K*_m_) are presented in Table 1. These results reveal that rat liver microsomes show around a 6-fold higher *V*_max_ value than human liver microsomes. *K*_m_ in incubations with rat microsomes was 4-fold lower than that in incubations with human microsomes. The unscaled CE for the rat data was around 24-fold higher than that for the human data. However, the scaled *V*_max_ in rats was 26-fold lower than the scaled *V*_max_ in humans amounting to 11.1 and 294 μmol/h, respectively. The scaled CE (calculated as scaled *V*_max_/*K*_m_) in rats and humans were 2.58 and 17.4 L/h, respectively.

**Table 1:** Kinetic Parameters for the Conversion of Aloe-Emodin to Rhein in Incubations with Rat or Human Liver Microsomes

	rats	humans
unscaled *V*_max_ (nmol/min/mg microsomal protein)	0.472	0.0786
*K*_m_ (μM)	4.31	16.9
unscaled CE^a^ (mL/min/mg microsomal protein)	0.110	0.00465
scaled *V*_max_^b^ (μmol/h)	11.1	294
scaled CE^c^ (L/h)	2.58	17.4

a Calculated as unscaled *V*_max_/*K*_m_.

b Calculated from unscaled (*in vitro*) *V*_max_ using a microsomal protein content of 46 mg microsomal protein/g liver for rats and 40 mg microsomal protein/g liver for humans.

c Calculated as scaled *V*_max_/*K*_m_.

**Figure 4:**
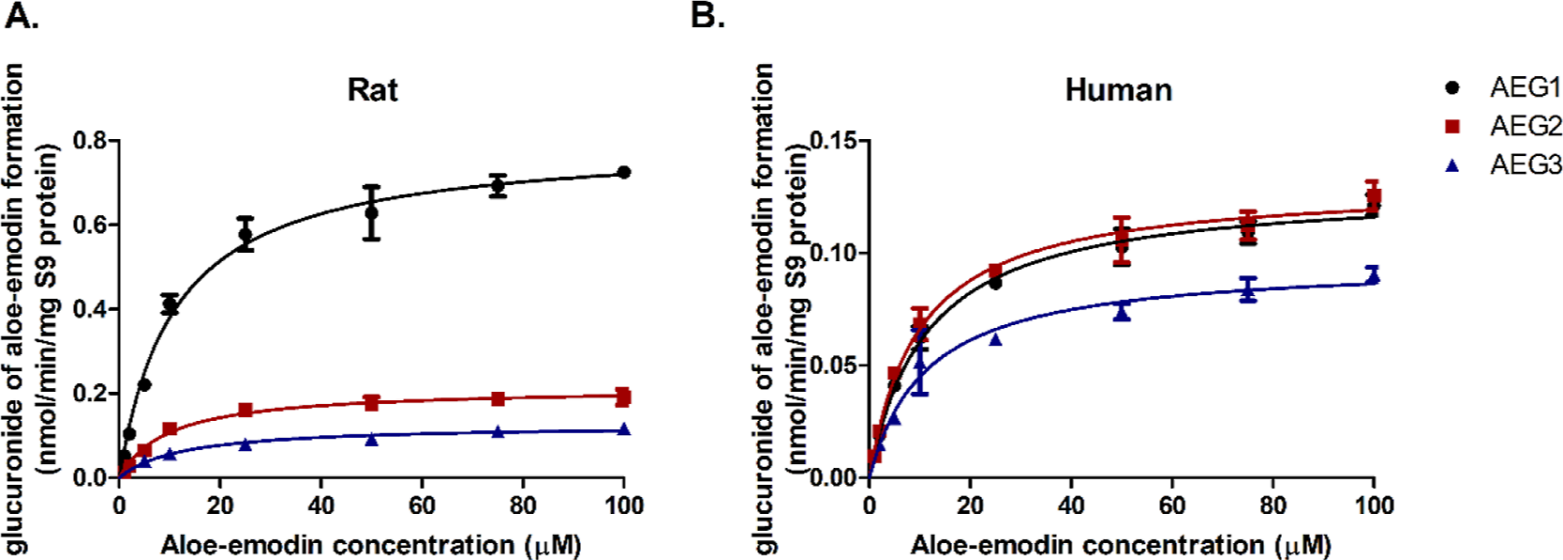
Concentration-dependent formation of aloe-emodin glucuronides including AEG1 (black circles), AEG2 (red squares) and AEG3 (blue triangles) in incubations with rat (A) and human (B) liver S9 fractions. Data points represent means ± SEM of three experiments for each conversion. Note the different *Y*-axis scale.

The kinetics of the glucuronidation of aloe-emodin were determined by *in vitro* incubations with pooled rat or human liver S9 fractions. Figure 4 shows the aloe-emodin concentration-dependent glucuronidation in rats and humans. The formation of aloe-emodin glucuronides was confirmed with β-glucuronidase hydrolysis, where, upon β-glucuronidase treatment, the peaks of the glucuronidated metabolites were reduced with a concomitant equimolar increase in the amount of aloe-emodin (Figure S4). The kinetic data (*V*_max_, *K*_m_ and CE) obtained for aloe-emodin glucuronides (AEGs) formation are summarized in Table 2 (rats) and Table 3 (humans). Three glucuronide metabolites (AEG1, AEG2, and AEG3), of which AEG1 is the major one formed in incubations with rat S9, exhibited an unscaled *V*_max_ that was 4-fold higher than that for AEG2 and 6-fold higher than the unscaled *V*_max_ for AEG3, while the *K*_m_ values for their formation were comparable (Table 2). In incubations with human liver S9, the same three glucuronide metabolites were formed. In this case, they exhibited comparable unscaled *V*_max_ and *K*_m_ values (Table 3), but their *V*_max_ values were lower than those observed in incubations with rat S9, while the *K*_m_ values were similar to the values in rats. Tables 2 and 3 also present the scaled *V*_max_ values which reveals that the scaled *V*_max_ values in humans were much higher than those in rats.

**Table 2:** Kinetic Parameters for Glucuronidation of Aloe-Emodin in Incubations with Rat Liver S9

	AEG1	AEG2	AEG3
unscaled *V*_max_ (nmol/min/mg S9 protein)	0.799	0.214	0.126
*K*_m_ (μM)	11.1	9.91	12.5
unscaled CE^a^ (mL/min/mg S9 protein)	0.0720	0.0216	0.0101
scaled *V*_max_^b^ (μmol/h)	67.2	18.0	10.6
scaled CE^c^ (L/h)	6.05	1.82	0.848

a Calculated as unscaled *V*_max_/*K*_m_.

b Calculated from unscaled (*in vitro*) *V*_max_ using a scaling factor of 165 mg S9 protein/g liver for rats.

c Calculated as scaled *V*_max_/*K*_m_.

**Table 3:** Kinetic Parameters for Glucuronidation of Aloe-Emodin in Incubations with Human Liver S9

	AEG1	AEG2	AEG3
unscaled *V*_max_ (nmol/min/mg S9 protein)	0.129	0.131	0.0961
*K*_m_ (μM)	11.4	9.78	11.4
unscaled CE^a^ (mL/min/mg S9 protein)	0.0113	0.0134	0.00841
scaled *V*_max_^b^ (μmol/h)	1459	1477	1086
scaled CE^c^ (L/h)	128	151	95.3

a Calculated as unscaled *V*_max_/*K*_m_.

b Calculated from unscaled (*in vitro*) *V*_max_ using a scaling factor 120.7 mg S9 protein/g liver for humans.

c Calculated as scaled *V*_max_/*K*_m_.

### 3.2 Hepatic Clearance of Rhein

**Figure 5:**
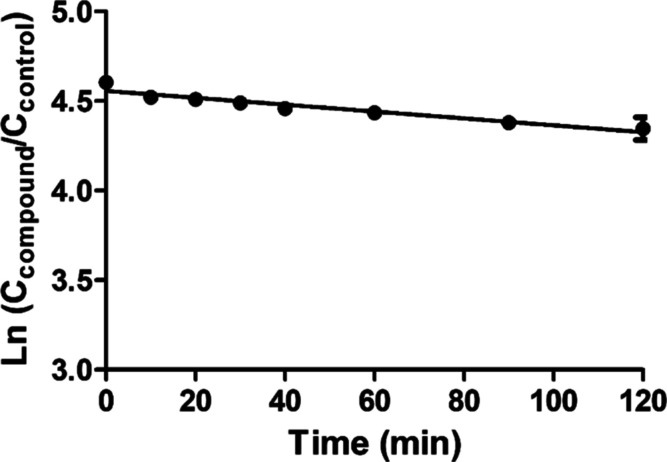
Time-dependent depletion of rhein in incubations with primary rat hepatocytes. Data points represent means ± SEM of three experiments.

Hepatic clearance of rhein in humans was set equal to 0 mL/min/million cells based on data reported for its conversion in incubations with primary human hepatocytes.^14^ For rat, no data were available, and the hepatic clearance of rhein was quantified in the present study using incubations of primary rat (male) hepatocytes with 1 μM rhein for 120 min (Figure 5). Based on the data obtained, the *in vitro* hepatic clearance of rhein for rats was calculated to be 0.00384 mL/min/million cells. Conversion to the *in vivo* situation resulted in a value for the rat *in vivo* hepatic clearance of rhein amounting to 0.264 L/h.

### 3.3 PBK Model Evaluation

The PBK models for aloe-emodin in rats and humans were established based on the kinetic parameters obtained in the *in vitro* incubations (Tables 1–3) and physiological and physicochemical input parameters (Tables S1 and S2). To evaluate the established models, model predictions were compared to reported *in vivo* data.

**Figure 6:**
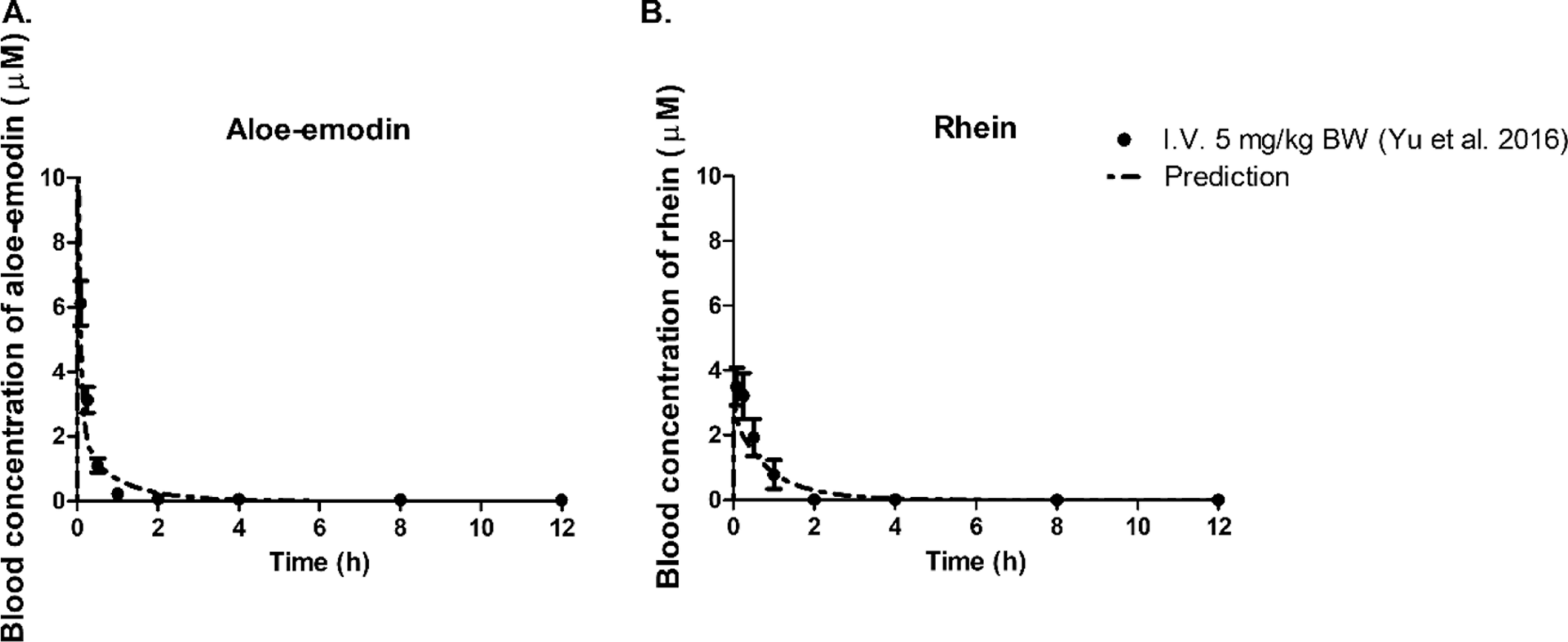
Comparison of time-dependent predicted blood concentration of aloe-emodin (A) and rhein (B) with reported *in vivo* data^8^ after a single intravenous (I.V.) dose (5.0 mg/kg BW) of aloe-emodin in rats within 12 h.

In the available *in vivo* studies, rats were intravenously or orally administered a single dose of aloe-emodin. Figure 6A,B presents the PBK model-predicted concentrations of aloe-emodin and rhein in blood compared with *in vivo* data after a single intravenous administration of aloe-emodin at 5.0 mg/kg BW. The results obtained indicate that the predictions match the experimental data well, indicating that the PBK model performed adequately. The PBK model performance was also evaluated using data obtained upon single oral doses of emodin (Table 4 and Figure S6). The reported *C*_max_ values of aloe-emodin and rhein following oral administration of 40 mg/kg BW and 300 mg/kg BW aloe-emodin were compared to the respective predicted *C*_max_ values, resulting in differences between the reported and predicted data which were 1.2–1.4 fold (Table 4 and Figure S6). This indicates that the rat PBK model could also adequately predict blood *C*_max_ values of aloe-emodin and the metabolite rhein upon oral administration. However, the predicted time-dependent plasma profiles deviate from the literature reported profiles, especially in terms of the *T*_max_ and the rate of clearance (Figure S6). Given that the model adequately predicts the concentration–time profiles and clearance upon I.V. dosing (Figure 6), the deviations observed upon oral dosing can best be ascribed to the aspects of intestinal absorption and/or to inaccuracy in the experimental *in vivo* data. Comparison of the two oral data sets for aloe-emodin in Figure S6A,B to one another indicates that the inaccuracy in the experimental data may likely explain the deviations. This follows from the fact that the concentration in the blood is reduced to less than 10% of *C*_max_ within 2 h in the data set of Yu *et al.*(8) (Figure S6A), while for the data set of Shi *et al.*(13) (Figure S6B), this extent of reduction has not been reached even after 12 h. Given this discrepancy in the oral data, further optimization of the model was carried out to fit the reported concentration time profiles upon oral dosing by modifying the rate constant *ka* for intestinal absorption to 4 h^–1^ and the % bioavailability, *Fa* to 0.022. With these parameters, the *C*_max_ was still predicted within 1.4 fold accuracy (Table S5), and the time-dependent concentration profile reported by Yu *et al.*(8) was well predicted, while the data reported by Shi *et al.*(13) were less well predicted than with the original data set (Figures S6 and S7). Given that both parameter sets predicted the *C*_max_ comparably well, the *ka* of 0.21 h^–1^ and *Fa* of 0.26 was derived by a method based on the Log *P*_app_ value obtained from the prediction *via* the pkCSM QSAR tool that also enabled definition of *ka* and *Fa* value for the human model. The human PBK model with *ka* = 0.14 h^–1^ and *Ka* = 0.36 was used for QIVIVE.

**Table 4:** Comparison of Predicted Maximum Blood Concentration (*C*_max_) Values of Aloe-Emodin and Rhein with Reported *In Vivo* Data after a Single Dose (40 mg/kg BW^8^ and 300 mg/kg BW^13^) Oral Administration of Aloe-Emodin in Rats (*ka* = 0.21 h^–1^, *Fa* = 0.26)

oral dose of aloe-emodin (mg/kg BW)	compound	reported *C*_max_	predicted *C*_max_	differences between predicted and reported data (fold)	references
40	aloe-emodin	0.10	0.12	1.2	(8)
300	aloe-emodin	0.70	0.99	1.4	(13)
40	rhein	0.83	1.00	1.2	(8)

### 3.4 *In Vitro* Concentration–Response Curves

**Figure 7:**
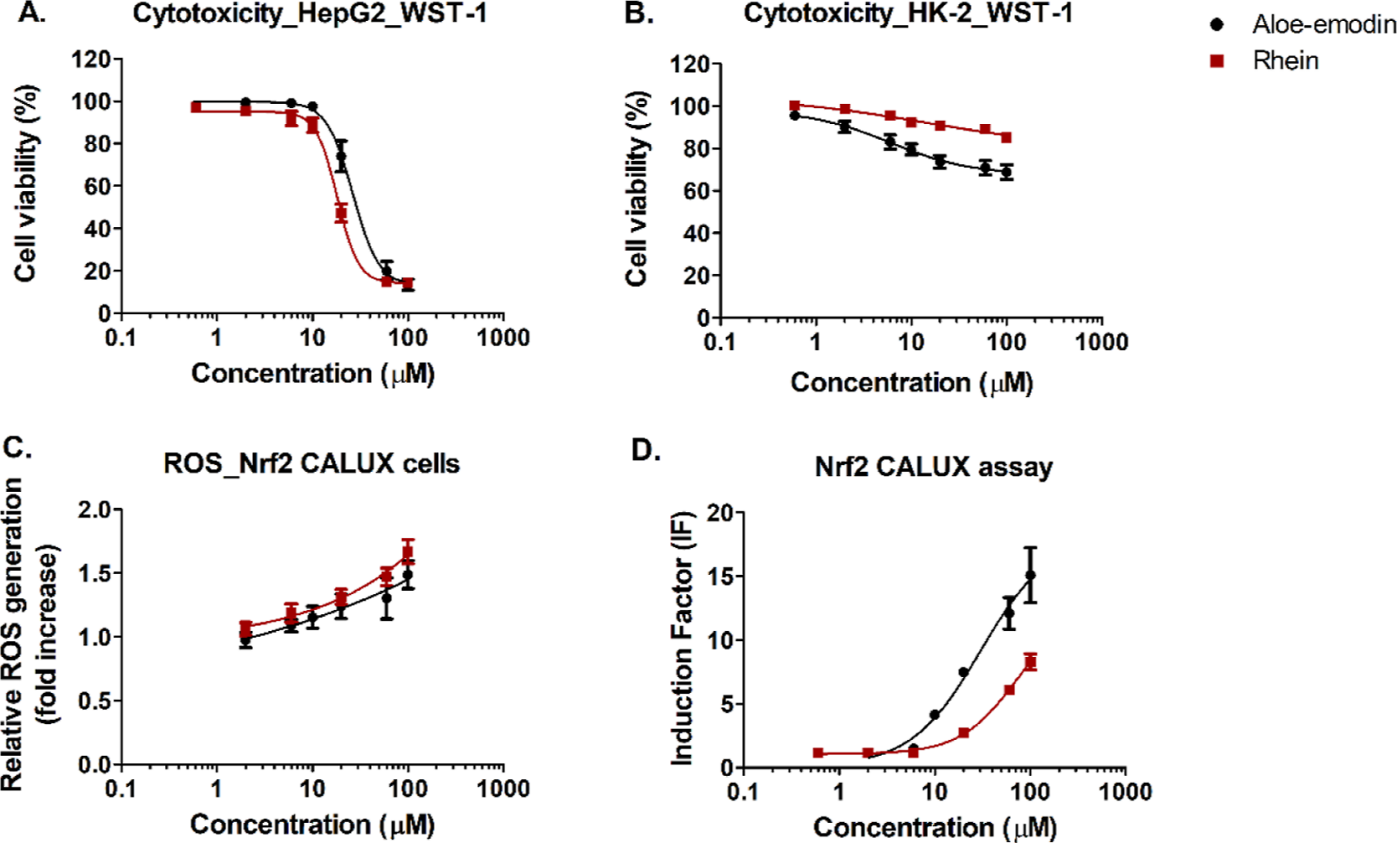
*In vitro* concentration–response curves of aloe-emodin (0.6–100 μM) and rhein (0.6–100 μM) for (A) cytotoxicity in HepG2 cells measured by the WST-1 assay, (B) cytotoxicity in HK-2 cells measured by the WST-1 assay, (C) ROS generation in the Nrf2 CALUX cells measured by the DCF-DA assay, and (D) Nrf2 activation by aloe-emodin and rhein measured by the Nrf2 CALUX reporter gene assay.^6^ Data points represent means ± SEM of at least three replicates.

Figure 7A,B shows the concentration–response curves for the cytotoxicity of aloe-emodin and rhein in HepG2 cells and HK-2 cells determined by the WST-1 assay. Figure 7C shows the concentration–response curves for ROS generation by aloe-emodin and rhein in Nrf2 CALUX cells, and Figure 7D shows the concentration–response curves for Nrf2 activation by aloe-emodin and rhein as quantified in the Nrf2 CALUX assay.^6^ With increasing concentrations of aloe-emodin and rhein, the viability of HepG2 cells decreased with EC_50_ values of 33.70 and 22.17 μM, and BMCL_10_ values of 7.3 and 6.3 μM, respectively, indicating that rhein is more toxic than aloe-emodin (Figure 7A). In kidney HK-2 cells, compared to aloe-emodin, rhein showed less cytotoxicity, with BMCL_10_ values amounting to 0.37 and 9.9 μM for aloe-emodin and rhein, respectively (Figure 7B). Due to the limited solubility of both aloe-emodin and rhein, concentrations exceeding 100 μM could not be tested. In addition to cytotoxicity, aloe-emodin and rhein show ROS generation in Nrf2 CALUX cells with BMCL_10_ values amounting to 0.1 and 0.16 μM, respectively, showing that aloe-emodin has a relatively higher potency for ROS generation than rhein (Figure 7C). Figure 7D shows the concentration-dependent Nrf2 activation by aloe-emodin and rhein with a BMCL_10_ amounting to 1.1 and 2.3 μM indicating the potency of aloe-emodin to be higher than that of rhein. The range of concentrations of aloe-emodin and rhein tested in the Nrf2 CALUX assay did not show cytotoxicity in the Nrf2 CALUX cells.^6^

Table 5 summarizes the BMCL_10_ and BMCU_10_ values of emodin and rhein derived from the data described above. Based on the BMCL_10_ values, the RPF values for rhein compared to aloe-emodin (RPF defined at 1.0) were defined for the cytotoxicity for HepG2 cells, HK-2 cells, ROS generation, and Nrf2 activation, enabling conversion of the concentrations of aloe-emodin and rhein to concentrations expressed in aloe-emodin equivalents by using these RPF values. The RPF of rhein amounted to 1.20 for hepatotoxicity, 0.037 for nephrotoxicity, 0.63 for ROS generation, and 0.48 for Nrf2 activation.

**Table 5:** BMCL_10_ and BMCU_10_ Values and RPF Values of Aloe-Emodin and Rhein for Cytotoxicity of HepG2 Cells, Cytotoxicity of HK-2 Cells, and Nrf2 Activation

end point	aloe-emodin BMCL_10_/BMCU_10_ (μM)	rhein BMCL_10_/BMCU_10_ (μM)	aloe-emodin RPF	rhein RPF^a^
cytotoxicity_HepG2	7.3/18.6	6.3/13	1.0	1.2
cytotoxicity_HK-2	0.37/5.2	9.9/42.7	1.0	0.037
ROS generation	0.1/53.5	0.16/14.6	1.0	0.63
Nrf2 activation	1.1/3.6	2.3/5.9	1.0	0.48

a Calculated based on BMDL_10_ value for each end of point.

### 3.5 Evaluation of Predicted *In Vivo* Dose-Dependent Response

**Figure 8:**
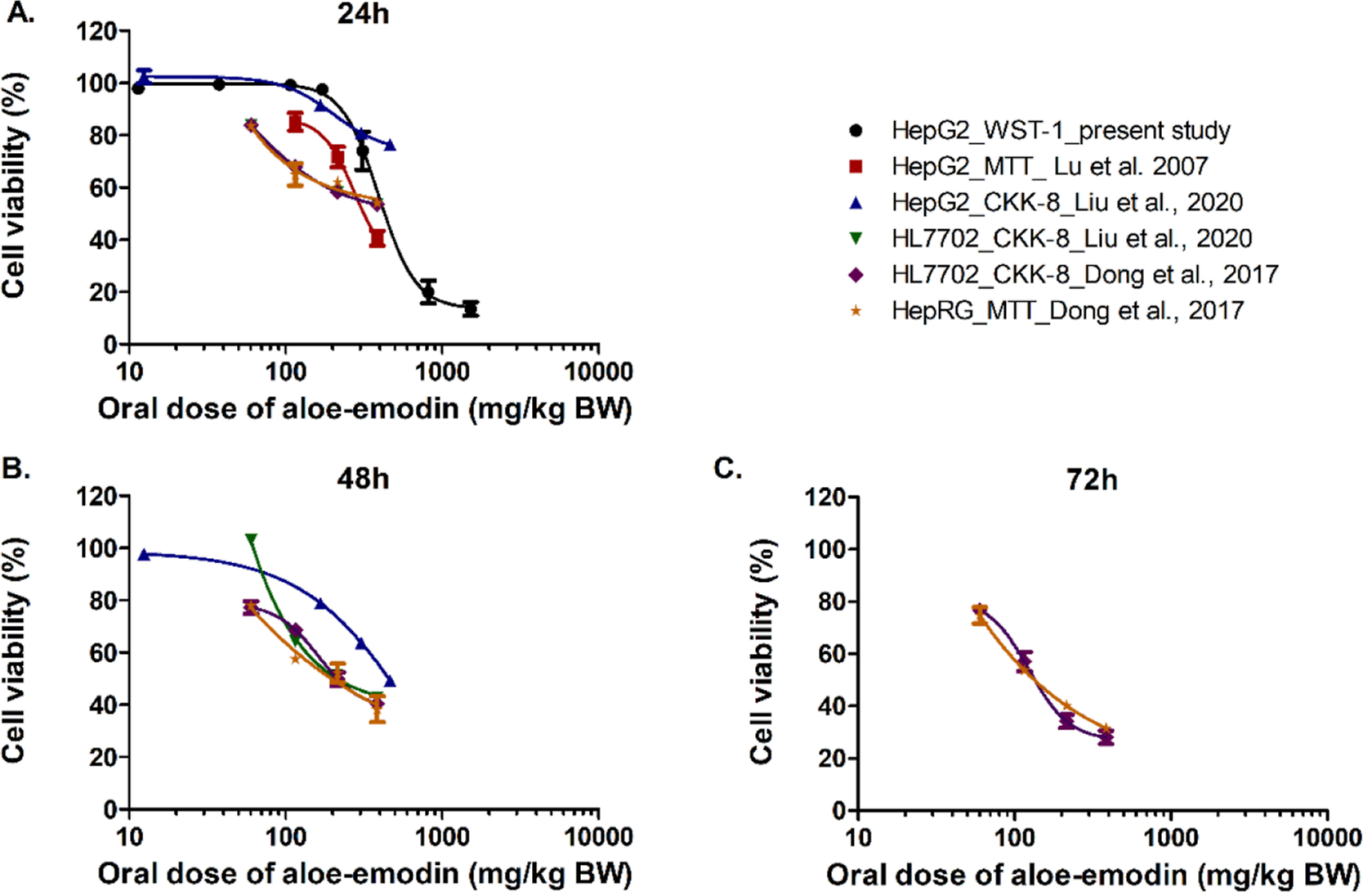
Predicted *in vivo* dose–response curves for hepatotoxicity obtained by PBK modeling-facilitated QIVIVE of the *in vitro* concentration–response curves for hepatotoxicity derived in the present study and from reported data after 24 h (A); 48 h (B), and 72 h (C) treatment of the cells with aloe-emodin.

**Figure 9:**
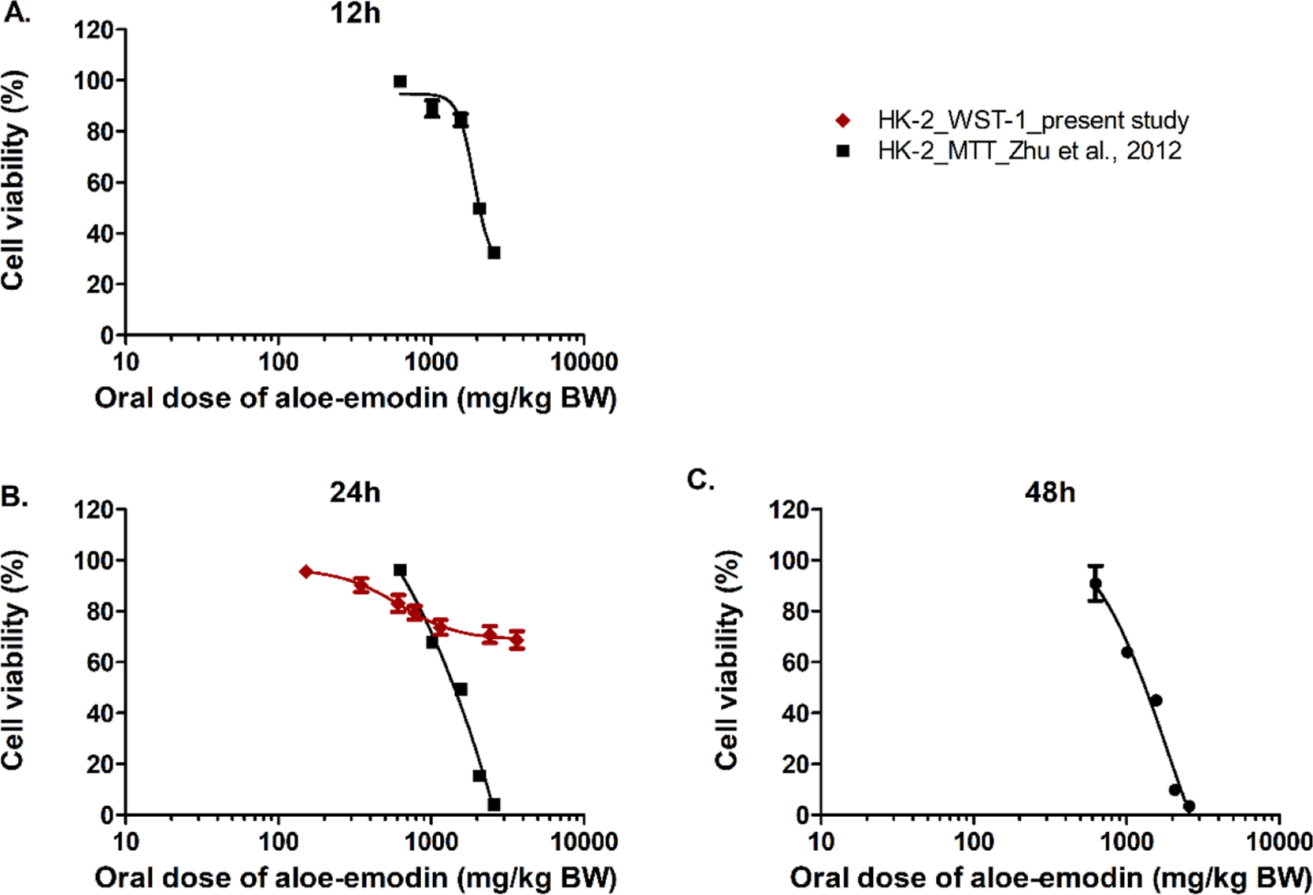
Predicted *in vivo* dose–response curves for nephrotoxicity obtained by PBK modeling-facilitated QIVIVE of the *in vitro* concentration–response curves for nephrotoxicity derived in the present study and from reported data after 12 h (A); 24 h (B), and 48 h (C) treatment of the cells with aloe-emodin.

**Figure 10:**
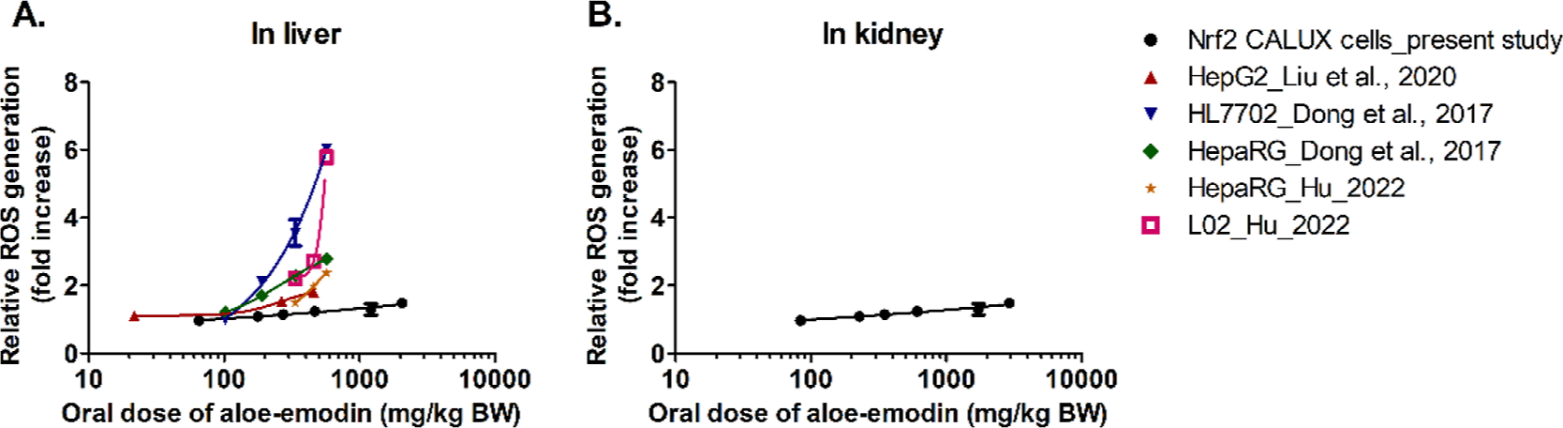
Predicted *in vivo* dose–response curves for ROS formation in liver (A) or kidney (B) obtained by PBK modeling-facilitated QIVIVE of the *in vitro* concentration–response curves for ROS formation determined in the present study or from reported data.

**Figure 11:**
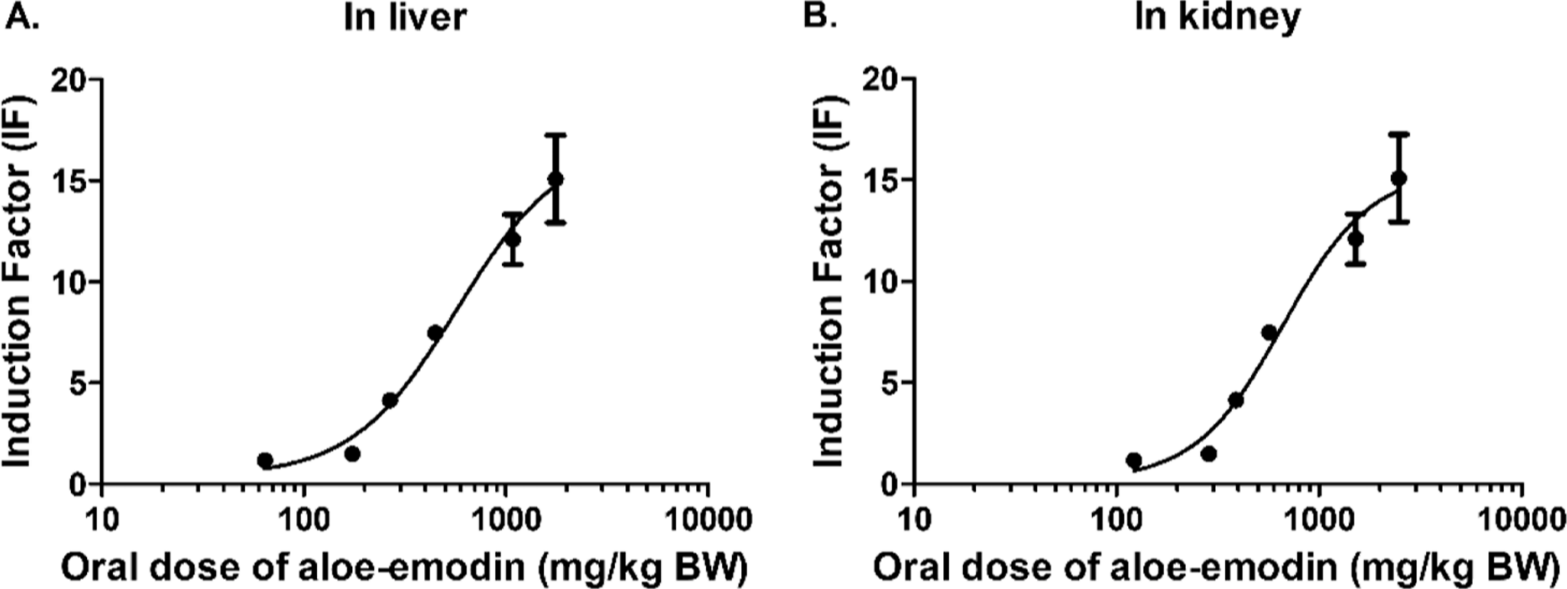
Predicted *in vivo* dose–response curves for Nrf2 activation in liver (A) and kidney (B) obtained by PBK modeling-facilitated QIVIVE of the *in vitro* concentration–response curves for Nrf2 activation derived from reported data after 24 h treatment of aloe-emodin.

Figures 8–11 show the predicted *in vivo* dose–response curves of aloe-emodin for hepatotoxicity, nephrotoxicity, ROS generation, and Nrf2 activation. These curves were converted from the concentration–response curves obtained in the respective *in vitro* assays (Figures S1–S3) using PBK modeling-facilitated QIVIVE. The QIVIVE included the contribution of rhein by converting its PBK model-predicted concentrations into aloe-emodin equivalents. Figure 8 presents the predicted *in vivo* dose–response curves for hepatotoxicity of aloe-emodin obtained by QIVIVE of the *in vitro* data in different cell lines. The results obtained reflect the variability also noted in the *in vitro* data (Figure S1). Predictions based on *in vitro* data from the less sensitive HepG2 cells resulted in higher predicted doses needed to induce *in vivo* hepatotoxicity than what was found based on data from the more sensitive HL7702 and HepaRG cells (Figure S1). Additionally, also in line with the *in vitro* data used as the basis for QIVIVE, the predicted *in vivo* dose–response curves shift to lower dose ranges with increasing exposure durations of the *in vitro* assays (Figure S1). Figure 9 presents the predicted *in vivo* dose–response curves for nephrotoxicity of aloe-emodin obtained by QIVIVE of the reported *in vitro* data sets on the cytotoxicity of aloe-emodin in HK-2 cells (Figure S2). Comparison of the *in vivo* dose–response curves in Figure 9 to those in Figure 8 indicates that for aloe-emodin, hepatotoxicity appears to be a more sensitive end point than nephrotoxicity. QIVIVE of *in vitro* data for the induction of ROS generation by aloe-emodin results in the dose–response curves depicted in Figure 10. It is of interest to note that also this induction of ROS in the liver appears to be a more sensitive end point than the induction of ROS in the kidney. In liver tissue (Figure 10A), induction of ROS generation by aloe-emodin was observed in a dose range similar to that inducing hepatotoxicity (Figure 8). Figure 11 presents the *in vivo* dose–response curves predicted for aloe-emodin-induced Nrf2 activation, again indicating the liver to be responsive already at lower dose levels than the kidney.

### 3.6 Derivation and Evaluation of PODs

**Figure 12:**
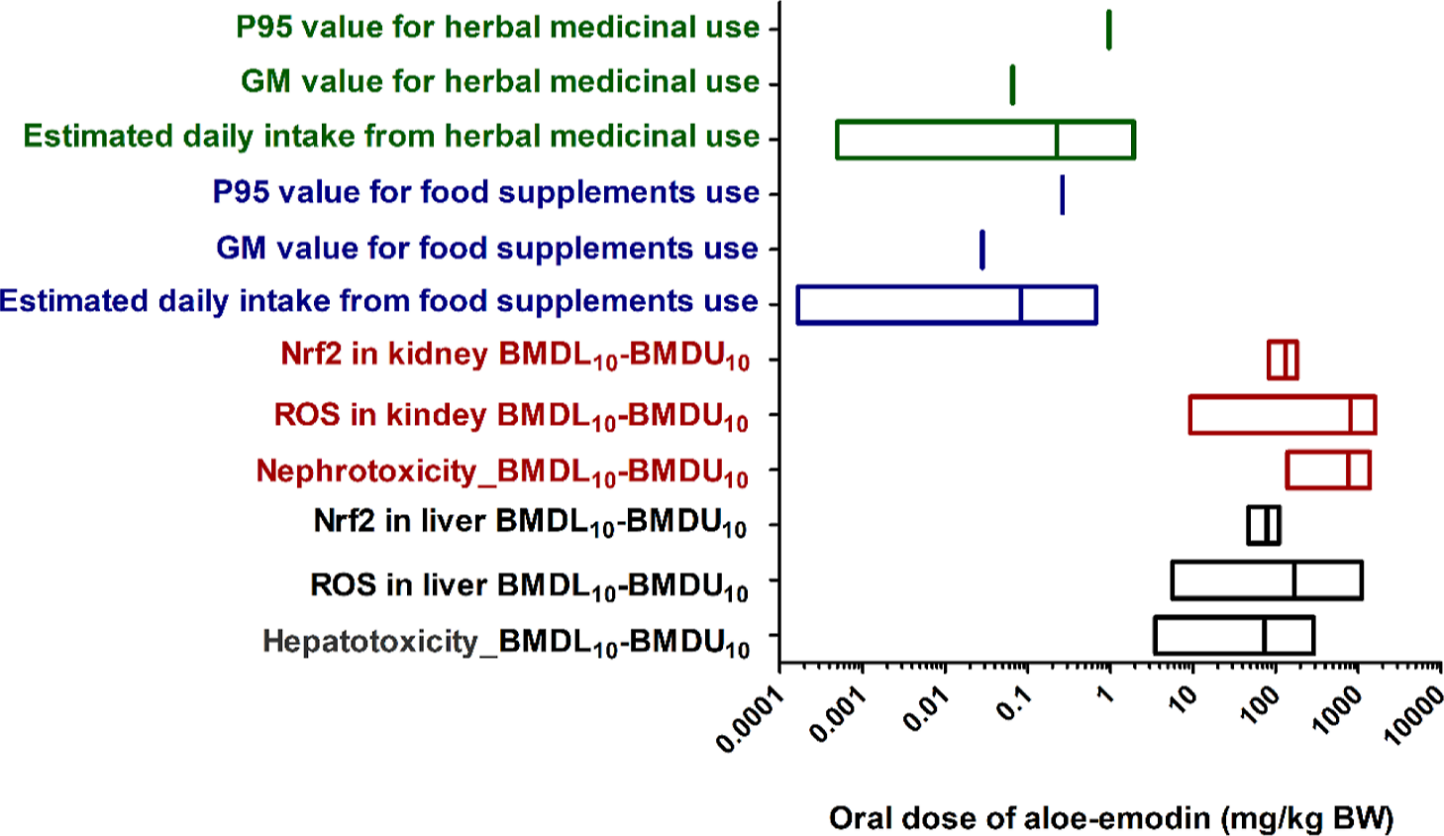
Comparison of predicted BMDL_10_–BMDU_10_ values derived from the predicted *in vivo* dose response curves by PBK modeling-facilitated QIVIVE, and the geometric mean (GM), 95th percentile (P95) and range of EDI of aloe-emodin resulting from consumptions of food supplements (blue) and herbal medicines (green) containing aloe-emodin and reported contents of aloe-emodin in respective botanicals. The BMDL_10_ and BMDU_10_ values were calculated based on each dose–response curve. Hepatotoxicity, nephrotoxicity, ROS generation, or Nrf2 activation in the liver and kidney are summarized and shown with different legends, respectively. The specific BMDL_10_ and BMDU_10_ values from each dose–response curve are shown in Table S6.

Using the *in vivo* concentrations in aloe-emodin equivalents thus obtained, the corresponding *in vivo* dose level of aloe-emodin (Figures 8–11) was calculated being the dose that would generate the unbound maximum venous blood concentration in the liver or kidney expressed in aloe-emodin equivalents (Figure S5). BMD analysis of these predicted dose–response curves was performed to derive BMDL_10_ and BMDU_10_ values, as illustrated in Figure 12 and summarized in Table S6. Specifically, the predicted BMDL_10_ value for hepatotoxicity is 3.5 mg/kg BW per day, which is lower than the BMDL_10_ values obtained for ROS generation (5.7 mg/kg BW) and Nrf2 activation (47 mg/kg BW) in the liver. However, in the kidney, the predicted BMDL_10_ value for nephrotoxicity is 140 mg/kg BW, which is higher than the predicted BMDL_10_ values for ROS generation (9.3 mg/kg BW) and Nrf2 activation (83 mg/kg BW). Compared with the BMDL_10_ values in the liver, the BMDL_10_ values in the kidney are higher. For instance, the BMDL_10_ for nephrotoxicity is 40-fold higher than that for hepatotoxicity, the BMDL_10_ for ROS generation in the kidney is 1.6 fold higher than that in the liver and the BMDL_10_ for Nrf2 activation in the kidney is 1.8 fold higher than that in the liver. These findings also suggest that the liver is more sensitive than the kidney to the toxic effects induced by aloe-emodin.

In addition, Figure 12 presents the EDIs of aloe-emodin from food supplements or herbal medicines. Based on the aloe-emodin content in various herbs summarized in Tables S3 and S4, supplemental usage is in line with recommendations from commercial food supplement suppliers and the Chinese Pharmacopoeia 2020 edition for medicines. The EDI of aloe-emodin from food supplement use varies from 0.00017 mg/kg BW per day to 0.68 mg/kg BW per day, while for medicinal use of aloe-emodin, the EDI of aloe-emodin varies from 0.0005 to 1.9 mg/kg BW per day (assuming a BW of 60 kg). The 95th percentile (P95) values of food supplements or herbal medicines are both lower than the lowest predicted BMDL_10_ value of hepatotoxicity, indicating a safety margin (BMDL_10_/EDI) of around 13-fold and 4-fold, respectively. Furthermore, the geometric mean for the EDI resulting from food supplement or herbal medicines is 126-fold and 53-fold lower than the predicted BMDL_10_ for hepatotoxicity, respectively.

## 4 Discussion

The aim of the present study was to predict the *in vivo* dose–response curves for the induction of hepatotoxicity, nephrotoxicity, ROS generation (in liver and kidney), and Nrf2 activation (in liver and kidney) by aloe-emodin, taking the activity of its metabolite rhein into account. The PBK modeling-facilitated QIVIVE approach, as an alternative to animal testing, was applied to convert *in vitro* concentration–response curves to *in vivo* dose–response curves for aloe-emodin induced hepatotoxicity, nephrotoxicity, ROS generation, and Nrf2 activation in humans. PODs were obtained by BMD analysis of the predicted *in vivo* curves and compared to EDIs of aloe-emodin from food supplements and herbal medicines, which are unlikely to result in the induction of hepatotoxicity, nephrotoxicity, ROS generation, or Nrf2 activation in the liver and kidney.

Aloe-emodin is reported to exert a variety of biological activities; however, its bioavailability has been shown to be quite low due to poor intestinal absorption,^37^ which is in agreement with the fraction of the dose absorbed (*Fa*) and the rate constant for intestinal uptake (*ka*) predicted by *in silico* calculations.^17,19,21,22^ In addition to oral administration, dermal exposure to aloe-emodin *via Aloe vera* gel for skincare is another main exposure route. *In vivo* studies (mouse) did not show any discernible effects on the liver and kidney upon dermal exposure of aloe-emodin at 10 and 1 mg/mL twice daily for 30 continuous days,^38^ and no effects on BWs or survivals upon dermal exposure of aloe-emodin at 7.46 or 74.6 μg/g every 5 days per week for 40 weeks.^39^ In addition, no skin permeability data are available. Thus, percutaneous absorption is not considered in our models and predictions. Our investigations exclusively focused on the oral administration of aloe-emodin by taking food supplements or herbal medicines.

Some species differences in the toxicokinetics of aloe-emodin and rhein were observed in our study. Rats are more efficient in the biotransformation of aloe-emodin than humans (Figures 3, 4, Tables 1, 2 and 3). This discrepancy may be attributed to species differences in the activities of CYP3A4 which is the primary metabolic enzyme for aloe-emodin.^40^ Notably, CYP3A4 was reported to show higher enzymatic activity in rat liver microsomes than in human liver microsomes.^41^ Given the role of CYP3A4 in the conversion of aloe-emodin to rhein, one could also expect that the interindividual variability in CYP3A4 activity within the human population may influence the bioactivation of aloe-emodin to rhein and the resulting toxicity. In the present study, the kinetics for the microsomal clearance of aloe-emodin and its conversion to rhein were quantified using pooled human liver microsomes. Future studies could quantify the effect of variation in CYP-mediated clearance and bioactivation of aloe-emodin by combining our PBK modeling with Monte Carlo simulations that select the respective kinetic parameters from the distribution describing the probability of the values for *V*_max_ and *K*_m_ within the human population.^42^ The sensitivity analysis presented in the present study reveals that especially variability in *V*_max_ and *K*_m_ for conversion of aloe-emodin to rhein will be of influence (Figure S8).

The hepatic clearance of rhein, whether in humans or in rats, derived from *in vitro* incubations with hepatocytes, is lower than that obtained from *in vitro* incubations with liver microsomes or cytosols.^25^ This observation is likely to be explained as that the ionized form of rhein at physiological conditions cannot easily diffuse across cellular lipid membranes.^43^ As a result, the actual intracellular concentration could be lower than the nominal concentration, resulting in a lower clearance in incubations with primary hepatocytes than in studies with subcellular fractions. Our model predictions for *C*_max_ of rhein fit well with available data in rats, indicating that the hepatic clearance of rhein as modeled based on data obtained with primary hepatocytes in this study is adequate.

It is worth mentioning that after oral administration of aloe-emodin, the *in vivo* time–concentration curves reported in the literature show two peaks for the blood concentration of aloe-emodin and rhein (Figure S6). Though the second peak is lower than the first one, its existence might point at the occurrence of enterohepatic circulation.^13,44^ The present work focused on the initial *C*_max_, and the second peak brought by enterohepatic circulation in the blood concentration upon prolonged time intervals was not considered since *C*_max_ is a proper dose metric for reflecting *in vivo* effects following single exposure to aloe-emodin, and the initial *C*_max_ was well predicted without taking the enterohepatic circulation into account (Table 4). Furthermore, the molecular weight threshold for biliary excretion in rats ranges from 200 to 325 g/mol, while for humans, it ranges from 500 to 600 g/mol.^45^ The molecular weights of AEGs and rhein glucuronide are 446 and 460 g/mol, respectively,^8,46^ which are lower than the human threshold, indicating that enterohepatic circulation of these two compounds would occur in rats instead of in humans.

To assess the safety of aloe-emodin for human consumption taking into account its active metabolite, rhein, data from *in vitro* studies of aloe-emodin in diverse cell models quantifying different end points were collected from the literature (Figures S1–S3). In addition, the *in vitro* concentration–response data for hepatotoxicity, nephrotoxicity, ROS generation, and Nrf2 activation were also assessed in the present study.

For cytotoxicity assessment, the WST-1 assay was applied. As compared to the MTT assay, the WST-1 assay is simpler, cost-effective, and less sensitive to disturbance by redox active compounds, like phenolic compounds, as these chemicals themselves may reduce MTT.^47^ As shown in Figures 7B and S3, the *in vitro* concentration curves obtained in the present study for liver toxicity matched the literature data relatively well, while the data reflecting nephrotoxicity of aloe-emodin obtained in the present study showed some differences from the reported data^48^ (Figures 7B and S3). One potential factor contributing to this difference may be attributed to variations in the cell seeding density. The low cell seeding density reported in a previous study cannot form a monolayer under our experimental conditions after 24 h growth. The formation of a monolayer is pivotal for creating a consistent and physiologically relevant cellular environment, and the discrepancy in seeding densities could have consequential effects on the observed nephrotoxicity of aloe-emodin.

Moreover, translating *in vitro* data on toxicity, quantified in cell types representing different target organs, to the *in vivo* situation by PBK modeling-facilitated QIVIVE, provides information on the potential variability in the sensitivity of different target organs to the adverse effects of aloe-emodin. By doing so, the results of the present study demonstrate that for aloe-emodin, hepatotoxicity is predicted to be a more sensitive end point than nephrotoxicity. Additionally, ROS generation and Nrf2 activation in the liver are predicted to occur at comparable dose levels as induction of hepatotoxicity (Figures 8–11). Furthermore, using different models and end points can also provide insights into the mode of action underlying the toxicity of aloe-emodin in humans. For instance, HepG2 cells have been reported to show lower CYP3A4 activity than other hepatic cell models.^49^ This may explain the lower predicted *in vivo* hepatotoxicity based on *in vitro* data from this cell line compared to the results obtained using metabolically competent cells like the liver HepaRG cells, as the conversion of aloe-emodin to its more active metabolite, rhein, would be less efficient in the HepG2 than in the HepaRG cells (Figure 7A and Table 5).

In addition, it is also of interest to consider which *in vitro* end point should be selected for QIVIVE. In theory, an *in vitro* model that quantifies functional end points for liver or kidney cells (*i.e.*, specific end points for liver or kidney cell functions) might result in effects at lower concentrations than what will be obtained for an end point like cell death. Consequently, QIVIVE based on functional end points may produce a lower POD compared to a POD obtained when using end points based solely on cell viability. The WST-1 assay used in the present study detects mitochondrial activity and is indicative of the metabolic activity of cells, and a reduction in the WST-1 response could reflect dedifferentiation, *i.e.*, a loss of functional competence of the cells studied. It remains to be determined whether an *in vitro* model quantifying a readout focused on a specific (and tissue specific) liver or kidney cell function would indeed provide a more sensitive POD.

In general, the daily intake of aloe-emodin from herbal medicines is higher than that from food supplement use (Figure 12, Tables S3 and S4). However, in some exposure scenarios, the daily intake of aloe-emodin from food supplement use by humans in some scenarios could be higher (Figure 12, Tables S3 and S4) because the high content of aloe-emodin is in aloe species, and the recommended dose of *Aloe vera* as food supplements is usually set at a high level by suppliers. Although EFSA in 2018 indicated that aloe-emodin was regarded as genotoxic and carcinogenic,^50^ a recent study indicated that aloe-emodin did not show positive results in an *in vivo* comet assay for kidney and colon cells in mice.^51^ This possible genotoxicity observed in studies reported by EFSA could result from the aloe-emodin-mediated induction of ROS formation.^51^ Taking this into account, it is of interest to note that the EDIs of aloe-emodin are below the BMDL_10_ values for ROS generation predicted by our PBK modeling-facilitated QIVIVE approach. Additionally, the predicted BMDL_10_ value for ROS generation by aloe-emodin were also lower than the BMDL_10_ for Nrf2 activation, which could be explained as that aloe-emodin can induce Nrf2 activation by ROS formation.^6,33,40^

It is also relevant to note that humans have been using aloe-emodin mainly *via* botanical products, and that other hydroxyanthraquinones such as rhein, emodin, chrysophanol, and physcion are also known to be present in these aloe-emodin containing botanicals.^52^ Potential interactions between aloe-emodin and these hydroxyanthraquinones as well as other botanical ingredients may exist and affect the biokinetics.^53^ In future studies such mixture effects could be taken into account when applying NAMs in risk assessment for aloe-emodin containing botanical food supplemental products and herbal medicines. For example, the PBK models defined in this work can be easily accommodated by modifying kinetic parameters for mixture effects.

In conclusion, the application of the PBK modeling-facilitated QIVIVE approach provides an alternative method to study the potential *in vivo* protective and toxic effects of aloe-emodin in humans without the need for animal tests or human intervention studies, contributing to the 3R development for future chemical risk assessment. Aloe-emodin predictions by the rat PBK model were validated by comparison to the available *in vivo* kinetic data. The predicted *in vivo* dose–response curves revealed that estimated dose levels of aloe-emodin, resulting from food supplements or herbal medicines, are unlikely to result in induction of hepatotoxicity, nephrotoxicity, ROS generation, or Nrf2 activation in liver and kidney.

## Supporting Information Available

Materials and Methods; *In vitro* concentration–response curves for hepatotoxicity of aloe-emodin in liver cells; *In vitro* concentration–response curves for nephrotoxicity of aloe-emodin in kidney cells; *In vitro* concentration–response curves for aloe-emodin induced ROS generation; overlay of HPLC-UV chromatograms of incubations of aloe-emodin; PBK modeling-based predictions of dose-dependent maximum concentration of aloe-emodin; comparison of time-dependent predicted blood concentration of aloe-emodin and rhein; local sensitivity analysis for the predicted maximum blood concentration of aloe-emodin and rhein; physiological parameters used in the rat and human PBK models for aloe-emodin and rhein; physiochemical parameters of aloe-emodin and rhein in rats and humans; botanical source, content in food supplements, its recommended daily use dosage by suppliers, content of aloe-emodin in the botanicals, and the EDIs of aloe-emodin; comparison of predicted maximum blood concentration (*C*_max_) values; summary of *in vitro* data of aloe-emodin; and PBK modeling code for aloe-emodin (PDF)
