# Supplementary material for: Physiologically based Kinetic Modeling-Facilitated Quantitative In Vitro to In Vivo Extrapolation to Predict the Effects of Aloe-Emodin in Rats and Humans
Source: J Agric Food Chem. 2024 Jul 9;72(29):16163–76. doi: 10.1021/acs.jafc.4c00969 (PMC11273626; doi:10.1021/acs.jafc.4c00969)
Supplement: Supplementary file 1 — jf4c00969_si_001.pdf [file jf4c00969_si_001.pdf]

**Supporting information**

# Physiologically based kinetic modeling-facilitated quantitative *in vitro* to *in vivo* extrapolation to predict effects of aloe-emodin in rats and humans

Qiuhui Ren*, Jiaqi Chen, Sebastiaan Wesseling, Hans Bouwmeester, Ivonne M.C.M. Rietjens

Division of Toxicology, Wageningen University and Research, Stippeneng 4, 6708 WE, Wageningen, The Netherlands

Corresponding author:

Qiuhui Ren*

Division of Toxicology, Wageningen University and Research, Stippeneng 4, 6708 WE, Wageningen, The Netherlands

*Email address: qiuhui.ren@wur.nl

Fax number: +31 (0) 317 48 49 31

## Materials and Methods

### Chemicals and biological materials

Aloe-emodin, rhein, dimethyl sulfoxide (DMSO), *tert*-butyl hydroperoxide (t-BHP, 70% in water), 2',7'-dichlorofluorescein diacetate ($H_2$DCF-DA), reduced nicotinamide adenine dinucleotide phosphate (NADPH), alamethicin, uridine 5'-diphosphoglucuronic acid (UGPGA), Trizma® base (Tris), β-glucuronidase form *Helix pomatia*, acetonitrile (ACN, UPLC/MS grade), methanol (UPLC/MS grade) and phosphate-buffered saline were purchased from Sigma-Aldrich (Zwijndrecht, The Netherlands). Magnesium chloride hexahydrate ($MgCl_2 \cdot 6H_2O$), hydrochloric acid (HCl) and formic acid were purchased from VWR International (Amsterdam, The Netherlands). [3-(4-Iodophenyl)-2-(4-nitrophenyl)-2H-5-tetrazolio]-1,3-benzene disulfonate (WST-1) was obtained from Roche (Mannheim, Germany).

Pooled hepatocytes from male Sprague Dawley rats (non-plateable), primary hepatocyte thawing and plating supplements for animal suspension (CM3000), and primary hepatocytes maintenance supplements (CM4000) were purchased from Thermo Fisher (Landsmeer, The Netherlands). Rat liver microsomes (Sprague Dawley, male), human liver microsomes (pooled from 150 donors, mixed gender), Pooled rat (Sprague Dawley, male) and human liver S9 fractions (pooled from 150 donors, mixed gender) were purchased from Corning (Amsterdam, The Netherlands). Williams' Medium E (1×, without phenol red), Dulbecco's Modified Eagle Medium with Ham's Nutrient Mixture F-12 (1:1) (DMEM/F12), DMEM/F12 without phenol red, trypsin 0.05% EDTA, nonessential amino acids (NEAA), penicillin/streptomycin (P/S) and phosphate-buffered saline (PBS, pH 7.4), were ordered from Gibco (Carlsbad, California, USA). Fetal bovine serum (FBS) was provided by Invitrogen (Breda, The Netherlands).

### Quantification of aloe-emodin and its metabolite rhein by LC-MS/MS

The quantification of aloe-emodin and rhein was carried out by LC-MS/MS analysis with a Phenomenex Kinetex C18 column (1.7 µm, 2.1 × 50 mm; Phenomenex, Utrecht, The Netherland). The mobile phase A was water containing 0.1% formic acid and mobile phase B was acetonitrile containing 0.1% formic acid. The flow rate was 0.3 ml/min. The gradient applied was: 0-3 min, 0-50% B; 3- 10 min, 50-70% B; 10-12 min, 70-100% B; 12-15 min, 100% B; 15-15.5 min, 100-0% B; 15.5-22 min, 0% B. The column temperature was kept at 25 ºC and the auto-sampler at 4 ºC. For samples incubated with liver microsomes, the injection volume was 25 µL, and for samples incubated with rat hepatocytes, the injection volume was 10 µL.

Mass spectrometric analysis was performed using a triple quadrupole mass spectrometer (Shimadzu LCMS-8045, Shimadzu, Japan), fitted with an electrospray ionization (ESI) source. Multiple reaction monitoring (MRM) in positive and negative modes were used for aloe-emodin and rhein, respectively. The ions monitored for aloe-emodin were m/z

271.15 > 225.10 (CE -20.0), m/z 271.15 > 253.00 (CE -36.0), and m/z 271.15 > 241.05 (CE -19.0); and those for thein were m/z 283.20 > 211.10 (CE 28.0), m/z 283.20 > 182.20 (CE 42.0), and m/z 283.20 > 154.20 (CE 48.0). Aloe-emodin and rhein levels in the samples were quantified using calibration curves created using commercially available reference compounds.

**Quantification of aloe-emodin and its glucuronides by UPLC-PDA**

To quantify the concentration of aloe-emodin and its glucuronides, a UPLC Nexera series (Shimadzu, Kyoto, Japan) equipped with a Photodiode Array (PDA) detector was utilized. Aloe-emodin and its glucuronides were separated on a Phenomenex C18 column (50 × 2.1 mm, 1.7 μm). Mobile phase A was nanopure water with 0.1% TFA and mobile phase B was acetonitrile. The gradient was: 10-90% B for 0-9.00 min; 90% B for 9.00-10.00 min, 90%-10% B for 10.00-10.30 min; 10% B for 10.30-15.00 min. 5 μl of sample were loaded on the column and elution was performed with a flow rate of 0.3 ml/min. The temperature of the column was at 40 °C. At a wavelength of 225 nm, aloe-emodin and its glucuronides were quantified based on comparison of the respective peak areas to the peak areas of a linear calibration curve for aloe-emodin prepared in Tris-HCl pH 7.4 containing 20% (v/v) ACN reference standards.

**Calculation of kinetic parameters**

The conversion of aloe-emodin to rhein and the formation of glucuronidation from aloe-emodin in *in vitro* incubations with liver microsomes and liver S9 fractions were fitted to a standard Michaelis-Menten equation (Eq S1) to calculate kinetic parameters:

$$v = \frac{Vmax*[S]}{Km+[S]} \qquad \text{(Eq S1)}$$

in which [S] is the substrate concentration (μM), v is the rate of metabolite formation (nmol/min/mg microsomal protein or nmol/min/mg S9 protein), $V_{max}$ is the apparent maximum reaction rate (nmol/min/mg microsomal protein or nmol/min/mg S9 protein), along with an Michaelis-Menten constant ($K_m$, in μM).

To quantify the *in vitro* clearance rate ($CL_{int,\textit{in vitro}}$) for rhein, the remaining concentration of rhein in the incubations of rhein with rat hepatocytes ($C_{rhein}$) was compared to that in the corresponding incubation of rhein without rat hepatocytes as control sample ($C_{control}$) at each incubation time point. This allowed to derive the depletion curve of rhein against time, which was expressed as [ln ($C_{rhein}$ / $C_{control}$)]. The slope of the linear portion of the depletion curve represents the depletion rate constant (k, in $min^{-1}$), and the $CL_{int,\textit{in vitro}}$ was calculated using the following equation (Eq S2):

$$\text{CLint}, in\ vitro\ (\text{ml/min/million cells}) = \frac{k\ (\text{min}^{-1}) \times V\ (mL)}{n(million\ cells)} \qquad \text{(Eq S2)}$$

In which, k represents the depletion rate constant ($min^{-1}$), V the incubation volume (0.2 mL), and n the number of cells in the incubation (0.5 million cells).

As stated in section 3.2 in manuscript, the *in vitro* clearance of rhein with primary human hepatocytes was taken from literature where it was reported to be negligible amounting to 0 μL/min/million cells [1].

**Sensitivity analysis**

A local sensitivity analysis was conducted to assess the influence of individual parameters on the output of maximum concentration values of aloe-emodin or rhein from the PBK model. The normalized sensitivity coefficient (SC) was calculated with the following equation (Eq S3):

$$SC = \frac{(C' - C)}{(P' - P)} \times \frac{P}{C} \quad \text{(Eq S3)}$$

In which C is the initial value of the model output, C’ is the modified value of the model output with a 5% increase of an input parameter; P is the initial parameter value and P’ is the 5% increased input parameter value. Only one parameter was changed at a time. A parameter with an absolute value of SC greater than 0.1 is considered as an influential parameter on the output of the PBK modeling [2,3]. The larger the absolute value of SC, the higher the influence of the parameter on the model output. The sensitivity analysis was performed for $C_{max}$ as the parameter of interest and for a single oral administration of 40 or 300 mg/kg BW aloe-emodin, dose levels used in reported *in vivo* studies [4,5].

**Figures and Tables**

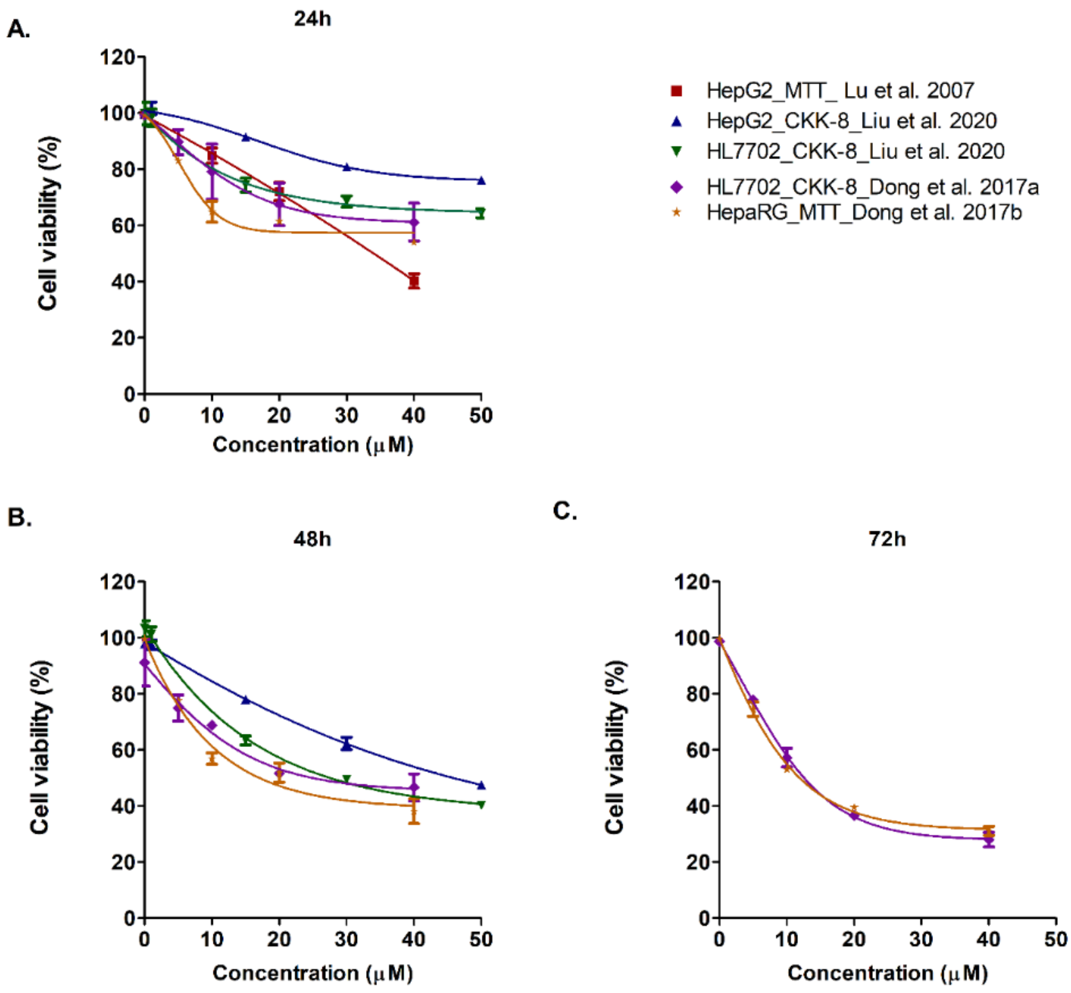


Figure S1. *In vitro* concentration-response curves for hepatotoxicity of aloe-emodin in liver cells (A) 24 h, (B) 48 h and (C) 72 h treatment as reported in literature [6–9].

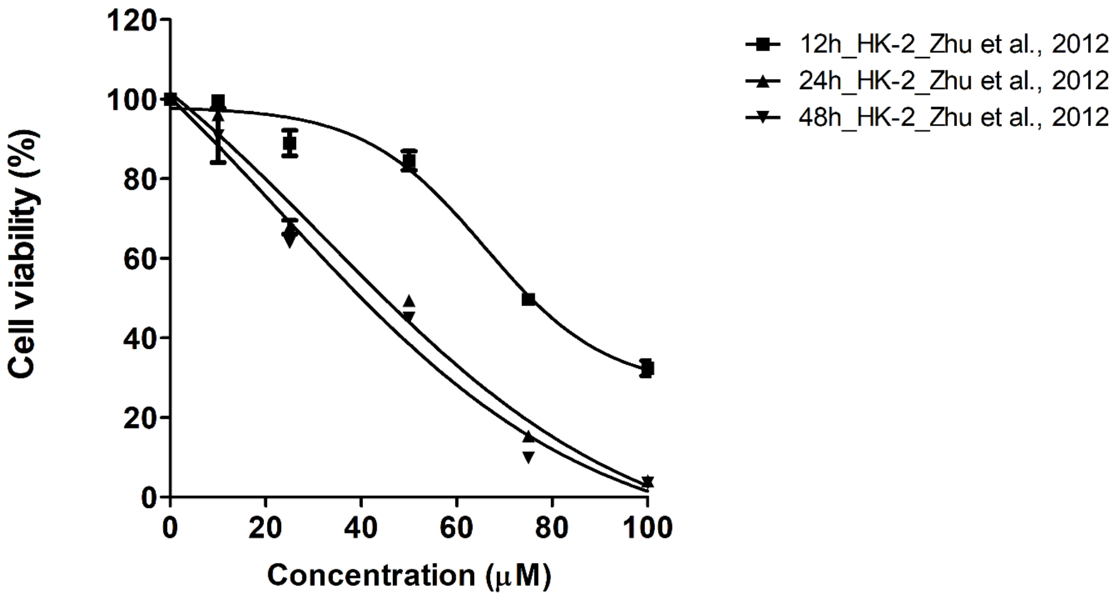


Figure S2. *In vitro* concentration-response curves for nephrotoxicity of aloe-emodin in kidney cells as reported in literature [10].

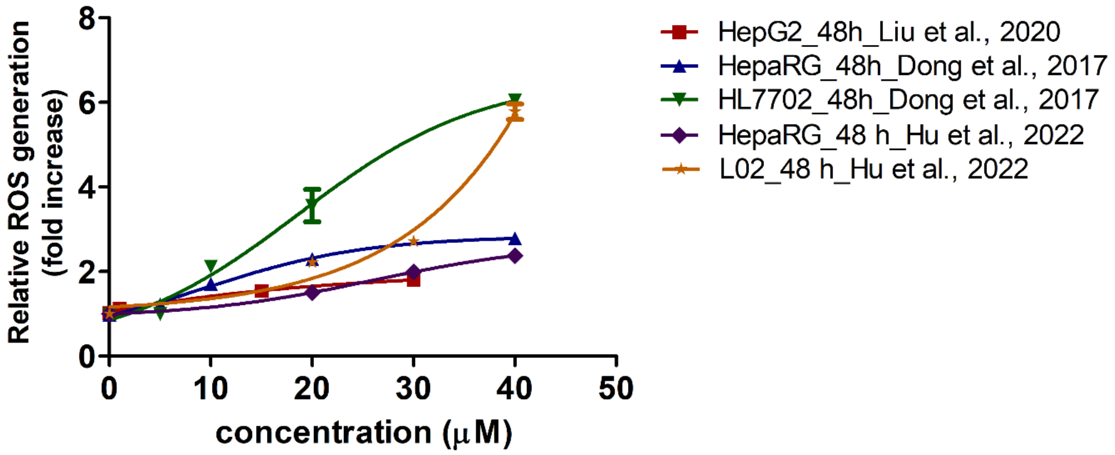


Figure S3. *In vitro* concentration-response curves for aloe-emodin induced ROS generation as reported in literature [6–8,11].

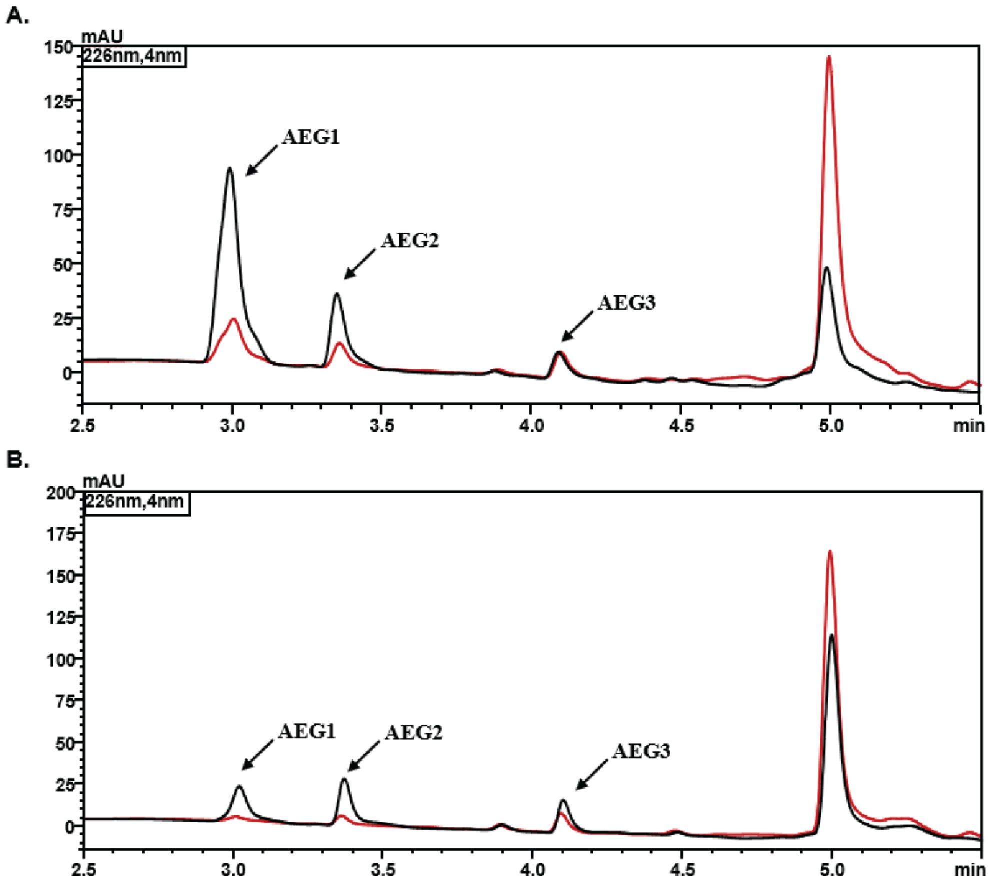


Figure S4. Overlay of HPLC-UV chromatograms of incubations of aloe-emodin with (A) rat liver S9 or (B) human liver S9 with (red) or without (black) beta-glucuronidase treatment.

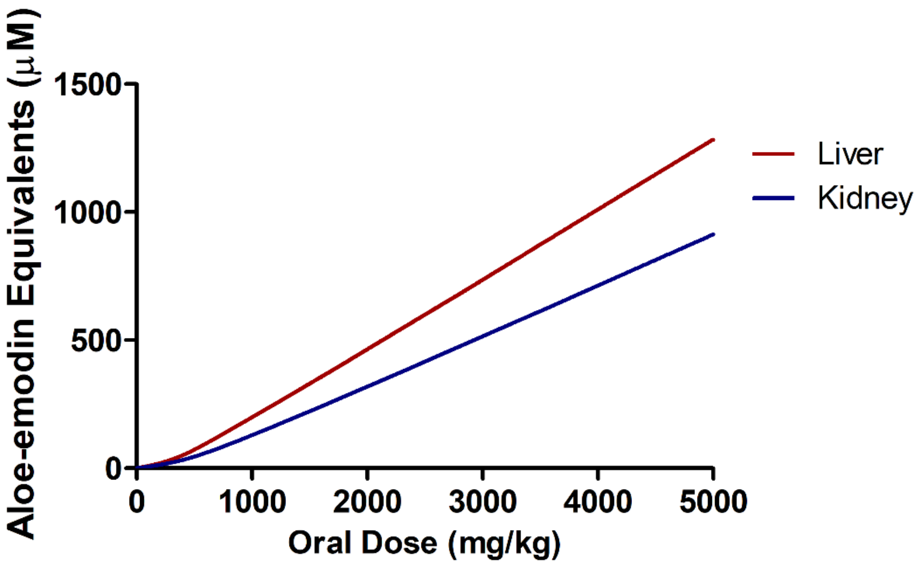


Figure S5. PBK modeling-based predictions of dose-dependent maximum concentration of aloe-emodin in venous blood of liver and venous blood of kidney used for QIVIVE.

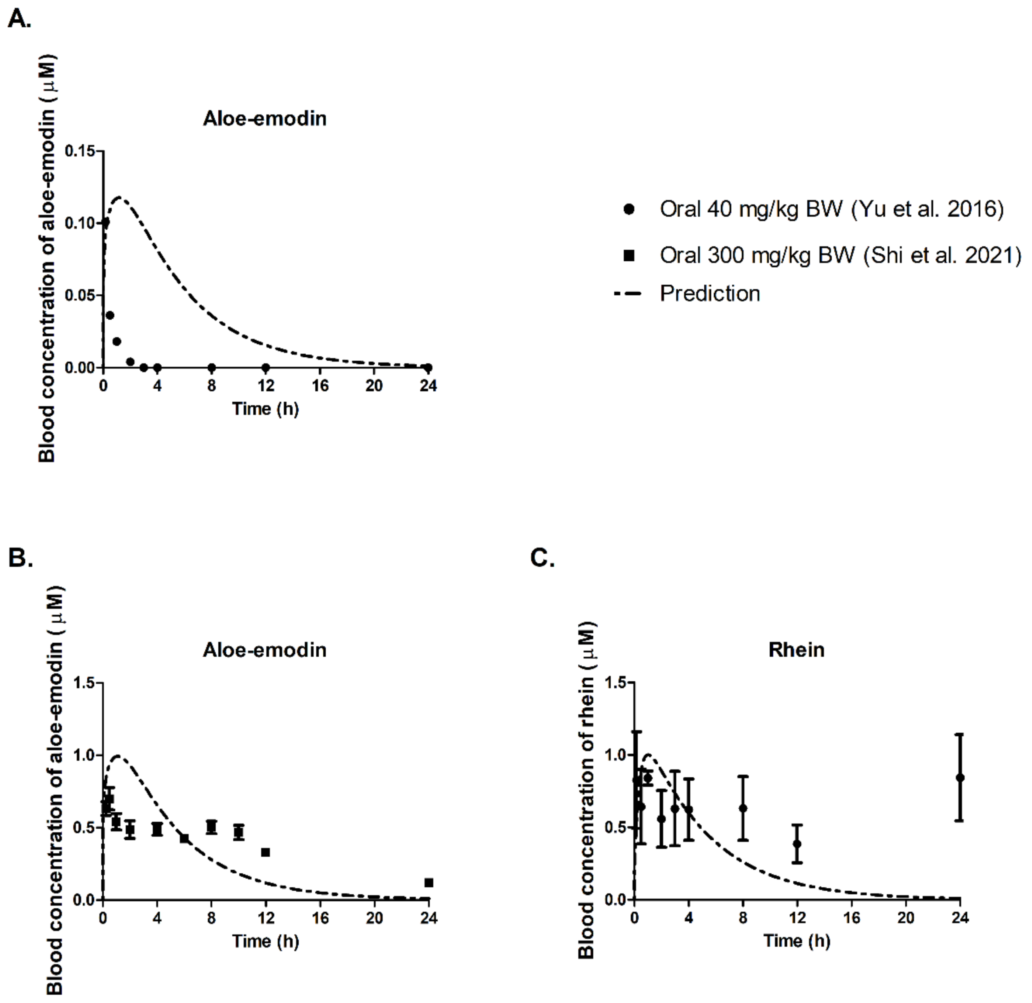


Figure S6. Comparison of time-dependent predicted blood concentration of aloe-emodin and rhein with reported *in vivo* data after a single (A. and C. 40 mg/kg [4]; B. 300 mg/kg [5]) oral administration of aloe-emodin in rats (ka = 0.21 $h^{-1}$; Fa = 0.26). Enterohepatic circulation was not included in the PBK model explaining part of the deviations. For further details see text in manuscript.

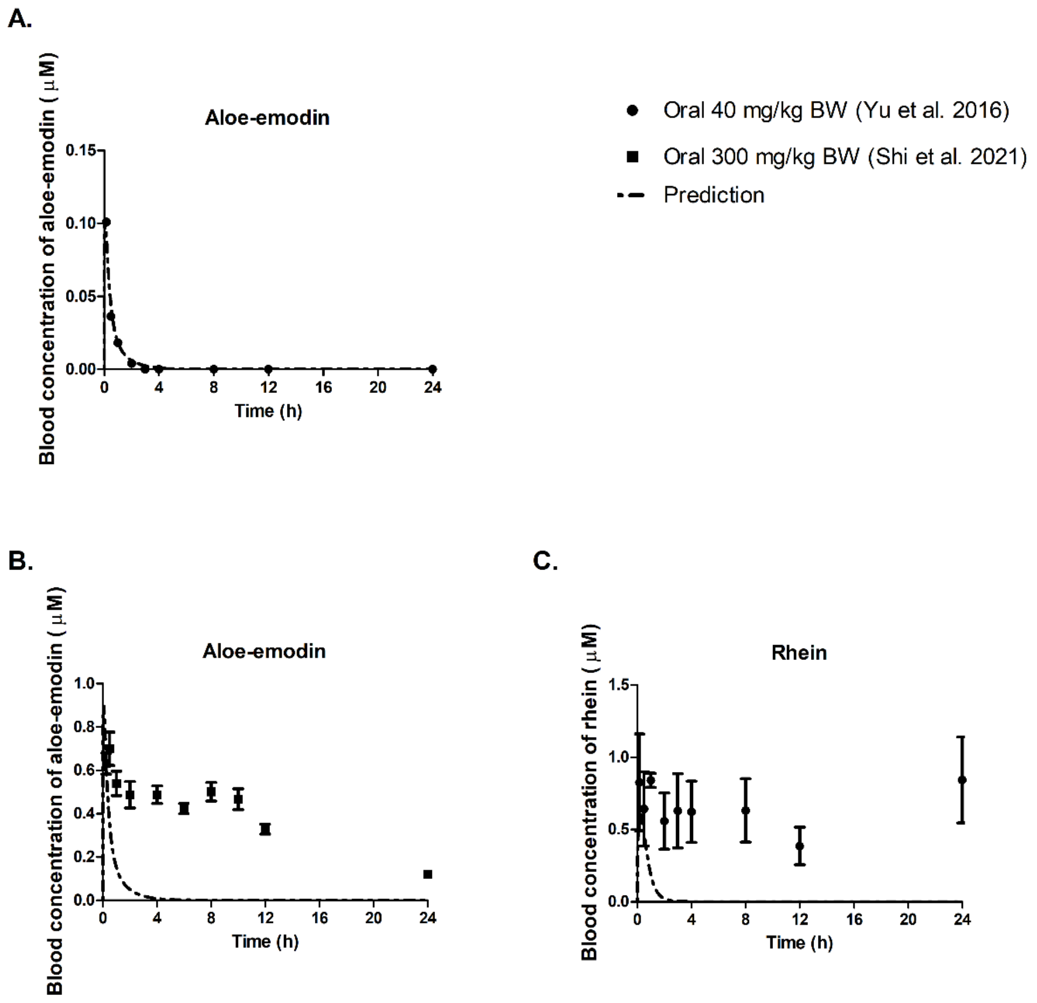


Figure S7. Comparison of time-dependent predicted blood concentration of aloe-emodin and rhein with reported in vivo data after a single (A. and C. 40 mg/kg [4]; B. 300 mg/kg [5]) oral administration of aloe-emodin in rats (ka = 4 $h^{-1}$; Fa = 0.022). Enterohepatic circulation was not included in the PBK model explaining part of the deviations. For further details see text in manuscript.

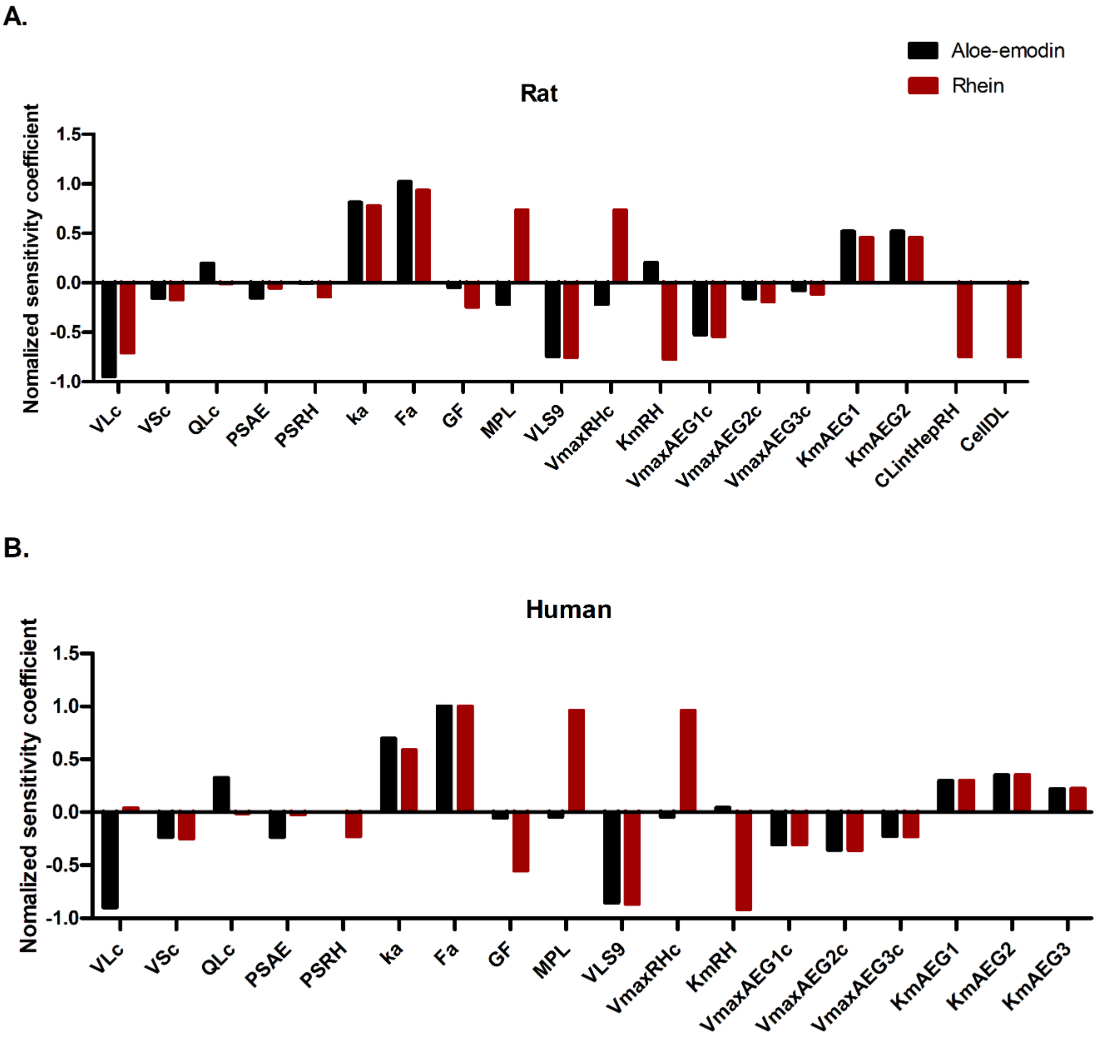


Figure S8. Local sensitivity analysis for the predicted maximum blood concentration of aloe-emodin and rhein in (A) rats and (B) humans after oral administration of aloe-emodin at a single dose of 40 mg/kg BW (rats) and 0.066 mg/kg BW (humans). Only model parameters with a normalized SC higher than 0.1 (absolute value) are shown. The parameters represent: VLc: fraction of liver tissue; VSc: fraction of slowly perfused tissue; QLc: fraction of blood flow to liver; PSAE: slowly perfused tissue:blood partition coefficient of aloe-emodin; PSRH: slowly perfused tissue:blood partition coefficient of rhein; ka: absorption rate constant of aloe-emodin from intestine to liver; Fa: fraction of dose absorbed; kb: bile excretion constant; GF: glomerular filtration rate; MPL: liver microsomal protein yield; VLS9: liver S9 protein yield; VmaxRHc maximum rate for conversion of aloe-emodin to rhein; KmRH: Michaelis-Menten constant for conversion of aloe-emodin to rhein; VmaxAG1c: maximum rate for conversion of aloe-emodin to AEG1; VmaxAG1c: maximum rate for conversion of aloe-emodin to AEG1; VmaxAG2c: maximum rate for conversion of aloe-emodin to AEG2; VmaxAG3c: maximum rate for conversion of aloe-emodin to AEG3; KmAG1: Michaelis-Menten constant for conversion of aloe-emodin to AEG1; KmAG2: Michaelis-Menten constant for conversion of aloe-

emodin to AEG2; KmAG3: Michaelis-Menten constant for conversion of aloe-emodin to AEG3; CLintHepRH: hepatic intrinsic clearance of rhein; CellDL: hepatocytes number in liver.

Note: Figure S8 shows the parameters with a normalized sensitivity coefficients higher than 0.1 obtained at 5% increase compared to their respective original values after oral administration of aloe-emodin at a single oral dose of 40 mg/kg BW in rat, a dose equal to one of the dose levels used in the reported *in vivo* studies [5] and a single oral dose of 0.066 mg/kg BW in human which is the geometric mean of daily exposure to aloe-emodin from medical use derived from reported data (Table S1). The results show that for both aloe-emodin and rhein in rat, fraction of liver (VLc), ka, Fa, liver S9 protein yield (VLS9), and the $V_{max}$ and $K_m$ for formation of AEG1 and AEG2 are the most influential parameters for prediction of blood concentrations of aloe-emodin and rhein while liver microsomal protein yield (MPL), and the $V_{max}$ and $K_m$ for conversion of aloe-emodin to rhein, $CL_{inthepRH}$ and hepatocytes number in liver (CellDL) have more influence on the predicted $C_{max}$ values for rhein than for aloe-emodin. In the human model, ka and Fa have substantial effects on the $C_{max}$ for both aloe-emodin and rhein. VLc shows a higher influence on the predicted $C_{max}$ for aloe-emodin while GF, MPL, $V_{max}$ and $K_m$ for rhein show a higher influence on the predicted $C_{max}$ for rhein. Different from the rat model, in human model $V_{max}$ and $K_m$ for conversion of aloe-emodin to AEG3 are also the influential parameters for prediction of blood concentration of aloe-emodin and rhein.

Table S1. Physiological parameters used in the rat and human PBK models for aloe-emodin and rhein [12,13].

| Model parameters | Rat | Human |
|---|---|---|
| **Physiological parameters** | | |
| Body weight (BW; kg) | 0.25 | 60.0 |
| **Percentage of body weight** | | |
| Liver | 3.4 | 2.6 |
| Fat | 7.0 | 21.4 |
| Kidney | 0.7 | 0.4 |
| Rapidly perfused tissues | 9.1 | 5.0 |
| Slowly perfused tissues | 72.4 | 58 |
| Blood | 7.4 | 7.9 |
| **Flow (L/h/kg $BW^{0.74}$)** | | |
| Cardiac output | 15 | 15 |
| **Percentage of cardiac output** | | |
| Liver | 25.0 | 22.7 |
| Fat | 9.0 | 5.2 |
| Kidney | 20.0 | 17.5 |
| Rapidly perfused tissues | 31.6 | 25.5 |
| Slowly perfused tissues | 14.4 | 29.1 |

Table S2. Physiochemical parameters of aloe-emodin and rhein in rats and humans, determined as described in Materials and Methods.

| | Aloe-emodin | Rhein |
|---|---|---|
| Molecular weight (g/mol) | 270.24 | 284.22 |
| LogP (predicted by ChemAxon) | 2.84 | 3.27 |
| PKa (predicted by ChemAxon) | 7.80; 8.46 | 3.40; 7.89; 8.54 |
| Blood plasma ratio | 0.55 | 0.95 (rat); 0.96 (human) |
| **Tissue:blood partition coefficients** | | |
| Fat | 6.42 | 0.14 |
| Liver | 1.89 | 0.33 (rat); 0.32 (human) |
| Kidney | 1.85 | 0.41 |
| Rapidly perfused tissues | 2.44 | 0.39 |
| Slowly perfused tissues | 2.77 | 0.34 |

Table S3. Botanical source, content in food supplements, its recommended daily use dosage by suppliers, content of aloe-emodin in the botanicals and the estimated daily intakes of aloe-emodin from food supplements use.

| **Botanical source** | **Botanical content** | **Recommended daily use dosage** | **Content of aloe-emodin (mg/g)** | **Estimated daily intakes (EDI) of aloe-emodin (mg/kg BW)** [a] | **References** |
|---|---|---|---|---|---|
| *Aloe barbadensis* (Leaves) | 20 g *Aloe vera* leaf extract [b] | 40g | 0.27 | 0.18 | 14 |
| | 10 g *Aloe vera* concentrate [c] | 10-20g | 0.27 | 0.045-0.09 | 14 |
| | *Aloe vera* juice 99.7% [d] | 30-90g | 0.27 | 0.14-0.41 | 14 |
| | 0.9 g of concentrated *Aloe vera* powder each capsule [e] | 0.9-1.8g | 0.27 | 0.0041-0.0081 | 14 |
| | aloe pure [f] | 40g | 0.27 | 0.18 | 14 |
| | 15 g *Aloe vera* extract per capsule [g] | 15-150g | 0.27 | 0.068-0.68 | 14 |
| *Rheum officinale* Baill. (Roots) | rhubarb 15 g/100 mL [h] | 1.5g | 0.23 | 0.0058 | 15 |
| | | | 0.65 | 0.016 | 16 |
| | | | 5.56 | 0.14 | 17 |
| | | | 0.6 | 0.015 | 23 |
| *Rheum palmatum* L. (Roots) | *rheum palmatum* 500mg/capsule [i] | 1-1.5g | 0.64-2.44 | 0.011-0.061 | 18 |
| | | | 0.59-3.28 | 0.0098-0.082 | 18 |
| | | | 2.03 | 0.034-0.051 | 19 |
| | | | 1.27 | 0.021-0.032 | 20 |
| | | | 0.58 | 0.0097-0.015 | 21 |
| | | | 0.01-7.71 | 0.00017-0.19 | 22 |
| | | | 0.6 | 0.01-0.015 | 23 |

[a] EDIs were calculated by "(Recommended daily use dosage as recommended by product supplier × Content of aloe-emodin)/Body weight (60 kg); [b] supplier: weightworld™; [c] supplier: NOW foods® ; [d] supplier: Holland&Barrett; [e] supplier: H&C Herbal Ingredients Expert; [f] supplier: Energetica Naura; [g] supplier: DeBa Pharma; [h] supplier: Swanson; [i] supplier: Dr.Giorgini Ser-Vis Srl.

Table S4. The botanical daily intake, the content of aloe-emodin in the botanicals and the estimated daily intakes of aloe-emodin from medicinal use.

| **Botanical source** | **Botanical daily intake (g/day)** [a] | **Content of aloe-emodin (mg/g)** | **Estimated daily intakes (EDI) of aloe-emodin (mg/kg BW)** [b] | **Reference** |
|---|---|---|---|---|
| *Aloe barbadensis* Miller (Leaves) | 2-5 | 0.27 | 0.009-0.023 | [14] |
| *Rheum officinale* Baill. (Roots) | 3-15 | 0.23 | 0.012-0.058 | [15] |
| | | 0.65 | 0.033-0.16 | [16] |
| | | 5.56 | 0.29-1.4 | [17] |
| *Rheum palmatum* L. (Roots) | | 0.64-2.44 | 0.032-0.12 | [18] |
| | | 0.59-3.28 | 0.030-0.82 | [18] |
| | | 2.03 | 0.10-0.51 | [19] |
| | | 1.27 | 0.064-0.32 | [20] |
| | | 0.58 | 0.029-0.15 | [21] |
| *Rheum palmatum/Rheum tanguticum* (Roots) | | 0.01-7.71 | 0.0005-1.93 | [22] |
| *Rheum palmatum* L., *Rheum tanguticum* Maxim. ex Balf. or *Rheum officinale* Baill. | | 0.60 | 0.03-0.15 | [23] |
| *Cassia obtusifolia* L. (Seeds) | 2-6 | 0.0714-0.4681 | 0.0024-0.047 | [24] |
| | | 0.697-2.078 | 0.023-0.21 | [25] |

[a] botanical daily intake was from Chinese Pharmacopoeia 2020 edition.

[b] EDIs were calculated by "(Botanical daily intake as recommended by the Chinese Pharmacopoeia (2020 edition) × Content of aloe-emodin)/Body weight (60 kg)".

Table S5. Comparison of predicted maximum blood concentration ($C_{max}$) values of aloe-emodin and rhein with reported *in vivo* data after a single dose (40 mg/kg BW [4] and 300 mg/kg BW [5]) oral administration of aloe-emodin in rats (ka = 4 $h^{-1}$; Fa = 0.022).

| **Oral dose of aloe-emodin (mg/kg BW)** | **Compound** | **Reported $C_{max}$** | **Predicted $C_{max}$** | **Differences between predicted and reported data (fold)** | **References** |
|---|---|---|---|---|---|
| 40 | Aloe-emodin | 0.10 | 0.10 | 1.0 | [4] |
| 300 | Aloe-emodin | 0.70 | 0.90 | 1.3 | [5] |
| 40 | Rhein | 0.83 | 0.61 | 1.4 | [4] |

Table S6. Summary of *in vitro* data of aloe-emodin in cell models and corresponding predicted $BMDL_{10}$ and $BMDU_{10}$ values obtained by using PBK modeling-facilitated QIVIVE.

| **Endpoint** | **Target organ** | **Cell type** | **Exposure duration (h)** | **Assay** | **Predicted $BMDL_{10}/BMDU_{10}$ (mg/kg BW per day)** | **References** |
|---|---|---|---|---|---|---|
| Hepatotoxicity | Liver | HepG2 | 24 | WST-1 | 132/289 | Present study |
| | | | 24 | MTT | 69.5/173 | [9] |
| | | | 24 | CKK-8 | 86.9/202 | [8] |
| | | | 48 | | 63.3/124 | |
| | Liver | HL7702 | 24 | CKK-8 | 59.5/127 | [8] |
| | | | 48 | | 17.6/56.1 | |
| | Liver | HepaRG | 24 | CKK-8 | 34.5/51.7 | [6] |
| | | | 48 | | 11.9/42.1 | |
| | | | 72 | | 23.5/55.4 | |
| | | | 24 | MTT | 6.83/53.5 | [7] |
| | | | 48 | | 3.52/43.3 | |
| | | | 72 | | 16.5/35.5 | |
| Nephrotoxicity | Kidney | HK-2 | 24 | WST-1 | 140/542 | Present study |
| | | | 12 | MTT | 983/1370 | [10] |
| | | | 24 | | 588/1030 | |
| | | | 48 | | 457/1030 | |
| ROS generation | Liver | Nrf2 CALUX cells | 4 | DCFDA | 5.65/1110 | Present study |
| | Kidney | | | | 9.34/1610 | |
| | Liver | HepG2 | 48 | DCFDA | 8.48/53.2 | [8] |
| | Liver | HepaRG | 48 | DCFDA | 44.1/74.4 | [7] |
| | Liver | | | | 162/191 | [11] |

| | | | | | | |
|---|---|---|---|---|---|---|
| | Liver | L02 | 48 | DCFDA | 72.1/181 | [11] |
| | Liver | HL7702 | 48 | DCFDA | 34.5/96.1 | [6] |
| Nrf2 activation | Liver | Nrf2 CALUX cells | 24 | Nrf2 CALUX assay | 47.2/111 | [26] |
| | Kidney | | | | 83.3/183 | [26] |

**PBK modeling code for aloe-emodin in rats**

```
;Date: September 2023

;Purpose: PBK model for Aloe-emodin

;Species: Rat (male)

;Compiled by: Qiuhui Ren and Jiaqi Chen

;Organization: Division of Toxicology, Wageningen University and Research, The Netherlands

;==============================================================

;Physiological parameters

;==============================================================

; tissue volume

BW = 0.250 {Kg} ; body weight rat (variable, depends on the study)

VFc = 0.070          ; fraction of fat tissue 12

VLc = 0.034          ; fraction of liver tissue 12

VKc = 0.007          ; fraction of kidney tissue 12

VBc = 0.074          ; fraction of blood 12

VRc = 0.091          ; fraction of rapidly perfused tissue 12

VSc = 0.724          ; fraction of slowly perfused tissue 12

                     ; total of fractions = 1

VF = VFc*BW          {L or Kg}     ; volume of fat tissue (calculated)

VL = VLc*BW          {L or Kg}     ; volume of liver tissue (calculated)

VK = VKc*BW          {L or Kg}     ; volume of kidney tissue (calculated)

VB = VBc*BW          {L or Kg}     ; volume of blood (calculated)

VR = VRc*BW          {L or Kg}     ; volume of rapidly perfused tissue (calculated)

VS = VSc*BW          {L or Kg}     ; volume of slowly perfused tissue (calculated)
```

;---------------------------------------------------------------------------------------------------------------

;blood flow

QC = 15*BW^0.74 ; cardiac output {L/hr} [12]

QFc = 0.090 ; fraction of blood flow to fat [13]

QLc = 0.250 ; fraction of blood flow to liver [13]

QKc = 0.200 ; fraction of blood flow to kidney [13]

QRc = 0.316 ; fraction of blood flow to rapidly perfused tissue [13]

QSc = 0.144 ; fraction of blood flow to slowly perfused tissue [13]

; total of fractions = 1

; Blood flow rates

QF = QFc*QC {L/hr} ; blood flow to fat tissue (calculated)

QL = QLc*QC {L/hr} ; blood flow to liver tissue (calculated)

QK = QKc*QC {L/hr} ; blood flow to kidney tissue (calculated)

QS = QSc*QC {L/hr} ; blood flow to slowly perfused tissue (calculated)

QR = QRc*QC {L/hr} ; blood flow to rapidly perfused tissue (calculated)

;=============================================================

;Physicochemical parameters

;=============================================================

;Tissue:plasma partition coefficients were predicted with QIVIVE tools (version 2.0) [27,28], then the tissue:blood partition coefficients used in the model were calculated by dividing the tissue:plasma partition coefficients by the blood plasma ratio (BPR), which was set as 0.55 for aloe-emodin as an acidic chemical when experimental data are unavailable [29].

;Aloe-emodin (AE) ; LogP 2.84; pKa1 7.80; pKa2 8.46 (Chemaxon)

BPRAE = 0.55 ; blood plasma ratio of aloe-emodin (assumed) [29]

fupAE = 0.092 ; fraction unbound in plasma of aloe-emodin (predicted by QIVIVE tools)

PFAE = 6.42 ; fat/blood partition coefficient

PLAE = 1.89 ; liver/blood partition coefficient

PKAE = 1.85 ; kidney/blood partition coefficient

PRAE = 2.44 ; rapidly perfused tissue/blood partition coefficient (brain gut heart lung spleen)

PSAE = 2.77 ; slowly perfused tissue/blood partition coefficient (bone muscle skin)

;Rhein (RH) ; LogP 3.27; pKa1 3.4; pKa2 7.89; pKa3 8.54 (Chemaxon)

BPRRH = 0.95 ; blood plasma ratio of rhein [30]

fupRH = 0.91 ; fraction unbound plasma of rhein [30]

PFRH = 0.14 ; fat/blood partition coefficient

PLRH = 0.33 ; liver/blood partition coefficient

PKRH = 0.41 ; kidney/blood partition coefficient

PRRH = 0.39 ; rapidly perfused tissue/blood partition coefficient (brain gut heart lung spleen)

PSRH = 0.34 ; slowly perfused tissue/blood partition coefficient (bone muscle skin)

;==============================================================

;Kinetic parameters

;==============================================================

; Transport from needle to blood

kn = 1000000 ; injection rate constant {/hr}

;------------------------------------------------------------------------------------------------------------------

; Absorption from GI-tract to liver

ka = 0.21 ; absorption rate constant {/hr}, (calculated, details in manuscript)

Fa = 0.26 ; fraction of dose absorbed (calculated, details in manuscript)

;------------------------------------------------------------------------------------------------------------

; Excretion from liver via bile

kb = 1 ; excretion rate constant {/hr} for biliary excretion [31]

; Excretion to urine via glomerular filtration

GF = 5.2 ; rat glomerular filtration rate {mL/min/kg bw} [32]

GFR = GF/1000*BW*60 ; scaled rat glomerular filtration rate {L/hr}

;------------------------------------------------------------------------------------------------------------

; Metabolism of aloe-emodin in liver

; MPL: rat liver microsomal protein yield scaling factor

MPL = 46 ; mg microsomal protein/g liver (QIVIVE tools [27])

; VLS9: rat liver S9 protein yield scaling factor

VLS9 = 165 ; mg S9 protein/g liver [33]

; Maximum rate for metabolism of aloe-emodin to rhein, measured in vitro in the present study

VmaxRHc = 0.4716 {nmol/min/mg microsomal protein}

; Scaled maximum rate in liver

VmaxRH = VmaxRHc/1000*60*MPL*VL*1000 {µmol/hr}

; Michaelis-Menten constant for metabolism of aloe-emodin to rhein, measured in vitro in the present study

KmRH = 4.314 {uM}

; Maximum rate for metabolism of aloe-emodin to aloe-emodin glucuronide 1 (AG1), aloe-emodin glucuronide 2 (AG2), and aloe-emodin glucuronide 3 (AG3), measured in vitro in the present study

VmaxAG1c = 0.7993 {nmol/min/mg S9 protein}

VmaxAG2c = 0.2135 {nmol/min/mg S9 protein}

VmaxAG3c = 0.1259 {nmol/min/mg S9 protein}

; Scaled maximum rate in liver

VmaxAG1 = VmaxAG1c/1000*60*VLS9*VL*1000 {µmol/hr}

VmaxAG2 = VmaxAG2c/1000*60*VLS9*VL*1000 {µmol/hr}

VmaxAG3 = VmaxAG3c/1000*60*VLS9*VL*1000 {µmol/hr}

; Michaelis-Menten constant for metabolism of aloe-emodin to aloe-emodin glucuronide 1 (AG1), aloe-emodin glucuronide 2 (AG2), and aloe-emodin glucuronide 3 (AG3), measured in vitro in the present study

KmAG1 = 11.05 {uM}

KmAG2 = 9.914 {uM}

KmAG3 = 12.52 {uM}

;-------------------------------------------------------------------------------------------------------------

; Hepatic clearance of rhein

CLintHepRH = 0.003836 ; (mL/min/million cells), hepatic clearance rate derived from in vitro incubations with rat hepatocytes in the present study

CellDL = 135*1000 ; (million cells/kg liver), hepatocyte number for rats = 1.35 *10E8 cells/g liver = 135 million cells/g liver = 135*1000 million cells/kg liver [34]

CLintRH = CellDL*VL*(CLintHepRH*60*1E-3) ; (L/hr), scaled hepatic clearance rate of rhein in rats

;============================================================

```
;Run settings

;==================================================================

;Molecular weight

MWAE = 270.24                   ; Molecular weight (g/mol) of aloe-emodin

MWRH = 284.22  ; Molecular weight (g/mol) of rhein



;oral dose

ODOSEmg = 40                                                  ; given oral dose in mg/kg bw

ODOSEumol2 = ODOSEmg*1E-3/MWAE*1E6          ; recalculated to umol/kg bw

ODOSEumol = ODOSEumol2*BW                 ; given oral dose in umol

;-------------------------------------------------------------------------------------------------------------------

;IV dose

IVDOSEmg = 0                                                  ; given IV dose in mg/kg bw

IVDOSEumol2 = IVDOSEmg*1E-3/MWAE*1E6        ; recalculated to umol/kg bw

IVDOSEumol = IVDOSEumol2*BW                           ; given IV dose in umol



;time

Starttime = 0                       ; in hr

Stoptime = 24                       ; in hr



;==================================================================

;Main model calculations: aloe-emodin (AE)

;==================================================================

;needle compartment
```

```
;ANe  = amount of AE in needle, umol

ANe'  = -kn*ANe

Init ANe = IVDOSEumol

;-------------------------------------------------------------------------------------------------------------------

;GI compartment

;AStAE  = amount of AE in GI tract, umol

AStAE'  = -ka*AStAE

Init AStAE = ODOSEumol*Fa

;-------------------------------------------------------------------------------------------------------------------

;liver compartment

;ALAE = amount of AE in liver tissue, umol

ALAE' = ka*AStAE + QL*(CBAE - CVLAE) - AbileAE' - AMLRH'-AMLG1'-AMLG2'-AMLG3'

Init ALAE = 0

CLAE = ALAE/VL

CVLAE = CLAE/PLAE


; AbileAE = amount of AE excreted from via bile

AbileAE' = kb*ALAE

Init AbileAE = 0


;AMLRH = amount of AE metabolized to rhein in liver

AMLRH' = (VmaxRH*CVLAE)/(KmRH + CVLAE)

Init AMLRH = 0
```

;AMLG1 = amount of AE metabolized to aloe-emodin glucuronide 1 (AG1) in liver, µmol

AMLG1' = (VmaxAG1*CVLAE)/(KmAG1 + CVLAE)

Init AMLG1 = 0

;AMLG2 = amount of AE metabolized to aloe-emodin glucuronide 2 (AG2) in liver, µmol

AMLG2' = (VmaxAG2*CVLAE)/(KmAG2 + CVLAE)

Init AMLG2 = 0

;AMLG3 = amount of AE metabolized to aloe-emodin glucuronide 3 (AG3) in liver, µmol

AMLG3' = (VmaxAG3*CVLAE)/(KmAG3 + CVLAE)

Init AMLG3 = 0

;-------------------------------------------------------------------------------------------------------------------

; kidney compartment

;AKAE = amount of AE in kidney tissue, umol

AKAE' = QK*(CBAE-CVKAE) -GFAE'

Init AKAE = 0

CKAE = AKAE/VK

CVKAE = CKAE/PKAE

;GFAE = amount of AE excreted to urine via glomerular filtration, µmol

GFAE' = GFR*CVKAE

Init GFAE = 0

;-------------------------------------------------------------------------------------------------------------------

;fat compartment

```
;AFAE = amount of AE in fat tissue, µmol

AFAE' = QF*(CBAE-CVFAE)

Init AFAE = 0

CFAE = AFAE/VF

CVFAE = CFAE/PFAE

;-----------------------------------------------------------------------------------------------------------------

;tissue compartment (rapidly perfused tissue)

;ARAE = amount of AE in rapidly perfused tissue, µmol

ARAE' = QR*(CBAE-CVRAE)

Init ARAE = 0

CRAE = ARAE/VR

CVRAE = CRAE/PRAE

;-----------------------------------------------------------------------------------------------------------------

;tissue compartment (slowly perfused tissue)

;ASAE = amount of AE in slowly perfused tissue, µmol

ASAE' = QS*(CBAE-CVSAE)

Init ASAE = 0

CSAE = ASAE/VS

CVSAE = CSAE/PSAE

;-----------------------------------------------------------------------------------------------------------------

; blood compartment

;ABAE = amount of AE in blood, µmol

ABAE' = kn*ANe + QF*CVFAE + QL*CVLAE + QK*CVKAE + QS*CVSAE + QR*CVRAE -
(QF+QL+QK+QS+QR)*CBAE
```

```
Init ABAE = 0

CBAE = ABAE/VB

CPAE = CBAE/BPRAE      ; plasma AE concentration


;=================================================================

;sub-model calculations: rhein (RH)

;=================================================================

; liver compartment

; ALRH = amount of RH in liver tissue, µmol

ALRH' = QL* (CBRH-CVLRH) + AMLRH'- AMintRH'

Init ALRH = 0

CLRH = ALRH/VL

CVLRH = CLRH/PLRH


; AMintRH = amount of RH cleared in liver

AMintRH' = CLintRH*CVLRH

Init AMintRH = 0

;------------------------------------------------------------------------------------------------------------------

; kidney compartment

;AKRH = amount of RH in kidney tissue, µmol

AKRH' = QK*(CBRH-CVKRH) -GFRH'

Init AKRH = 0

CKRH = AKRH/VK

CVKRH = CKRH/PKRH
```

;GFRH = amount of RH excreted to urine via glomerular filtration, umol

GFRH' = GFR*CVKRH

Init GFRH = 0

;-----------------------------------------------------------------------------------------------------------------

;fat compartment

;AFRH = amount of RH in fat tissue, µmol

AFRH' = QF*(CBRH-CVFRH)

Init AFRH = 0

CFRH = AFRH/VF

CVFRH = CFRH/PFRH

;-----------------------------------------------------------------------------------------------------------------

;tissue compartment (rapidly perfused tissue)

;ARRH = amount of RH in rapidly perfused tissue, µmol

ARRH' = QR*(CBRH-CVRRH)

Init ARRH = 0

CRRH = ARRH/VR

CVRRH = CRRH/PRRH

;-----------------------------------------------------------------------------------------------------------------

;tissue compartment (slowly perfused tissue)

;ASRH = amount of RH in slowly perfused tissue, µmol

ASRH' = QS*(CBRH-CVSRH)

Init ASRH = 0

CSRH = ASRH/VS

```
CVSRH = CSRH/PSRH

;----------------------------------------------------------------------------------------------------------------

; blood compartment

;ABRH = amount of RH in blood, µmol

ABRH' = QF*CVFRH + QL*CVLRH + QK*CVKRH + QS*CVSRH + QR*CVRRH -
(QF+QL+QK+QS+QR)*CBRH

Init ABRH = 0

CBRH = ABRH/VB

CPRH = CBRH/BPRRH     ; plasma RH concentration


;======================================================================

;Mass balance calculations of AE

;======================================================================

TotalAE = ODOSEumol*Fa + IVDOSEumol

CalculatedAE = ANe + AStAE + ALAE + AbileAE + AMLRH + AMLG1 + AMLG2 + AMLG3 + AKAE + GFAE +
AFAE + ASAE + ARAE + ABAE


ERRORAE = (TotalAE-CalculatedAE)/(TotalAE+1E-30)*100

MASSBBALAE = TotalAE-CalculatedAE + 1


;======================================================================

;Mass balance calculations of RH

;======================================================================

TotalRH = AMLRH
```

CalculatedRH = ALRH + AMintRH + AKRH + GFRH + AFRH + ASRH + ARRH + ABRH

ERRORRH = (TotalRH-CalculatedRH)/(TotalRH+1E-30)*100

MASSBBALRH = TotalRH-CalculatedRH + 1

**PBK modeling code for aloe-emodin in humans**

```
;Date: September 2023

;Purpose: PBK model for aloe-emodin

;Species: Human (mixed gender)

;Compiled by: Qiuhui Ren and Jiaqi Chen

;Organization: Division of Toxicology, Wageningen University and Research, The Netherlands

;==============================================================

; Physiological parameters

;==============================================================

; tissue volumes

BW = 60 {Kg}                ; body weight

VFc = 0.214                 ; fraction of fat tissue 12

VLc = 0.026                 ; fraction of liver tissue 12

VKc = 0.004                 ; fraction of kidney tissue 12

VBc = 0.079                 ; fraction of blood 12

VRc = 0.064                 ; fraction of rapidly perfused tissue 12

VSc = 0.613                 ; fraction of slowly perfused tissue 12

                            ; total of fractions = 1

VF = VFc*BW {L or Kg}       ; volume of fat tissue (calculated)

VL = VLc*BW {L or Kg}       ; volume of liver tissue (calculated)

VK = VKc*BW {L or Kg}       ; volume of kidney tissue (calculated)

VB = VBc*BW {L or Kg}       ; volume of blood (calculated)

VR = VRc*BW {L or Kg}       ; volume of rapidly perfused tissue (calculated)

VS = VSc*BW {L or Kg}       ; volume of slowly perfused tissue (calculated)
```

;-------------------------------------------------------------------------------------------------------------------

;blood flow rates [12]

```
QC = 15*BW^0.74          ; {L/hr}, cardiac output
QFc = 0.052              ; fraction of blood flow to fat
QLc = 0.227              ; fraction of blood flow to liver
QKc = 0.175              ; fraction of blood flow to kidney
QRc = 0.255              ; fraction of blood flow to rapidly perfused tissue
QSc = 0.291              ; fraction of blood flow to slowly perfused tissue
                         ; total of fractions = 1
QF = QFc*QC              {L/hr}   ; blood flow to fat tissue (calculated)
QL = QLc*QC              {L/hr}   ; blood flow to liver tissue (calculated)
QK = QKc*QC              {L/hr}   ; blood flow to kidney tissue (calculated)
QR = QRc*QC              {L/hr}   ; blood flow to rapidly perfused tissue (calculated)
QS = QSc*QC              {L/hr}   ; blood flow to slowly perfused tissue (calculated)
```

;=============================================================

; Partition coefficients

;=============================================================

;Tissue:plasma partition coefficients were predicted with QIVIVE tools (version 2.0) [27,28] ,then the tissue:blood partition coefficients used in the model were calculated by dividing the tissue:plasma partition coefficients by the blood plasma ratio (BPR), which was set as 0.55 for aloe-emodin as an acidic chemical when experimental data are unavailable [29]

;Aloe-emodin (AE)          ; LogP 2.84; pKa1 7.80; pKa2 8.46 (predicted by Chemaxon)

BPRAE = 0.55 ; blood plasma ratio of aloe-emodin (assumed) [29]

fupAE = 0.092 ; fraction unbound in plasma of aloe-emodin (predicted by QIVIVE tools)

PFAE = 6.42 ; fat/blood partition coefficient

PLAE = 1.89 ; liver/blood partition coefficient

PKAE = 1.85 ; kidney/blood partition coefficient

PRAE = 2.44 ; rapidly perfused tissue/blood partition coefficient (brain gut heart lung spleen)

PSAE = 2.77 ; slowly perfused tissue/blood partition coefficient (bone muscle skin)

;Rhein (RH) ; LogP 3.27; pKa1 3.4; pKa2 7.89; pKa3 8.54 (predicted by Chemaxon)

BPRRH = 0.96 ; blood plasma ratio of rhein [30]

fupRH = 0.91 ; fraction unbound in plasma of rhein [30]

PFRH = 0.14 ; fat/blood partition coefficient

PLRH = 0.32 ; liver/blood partition coefficient

PKRH = 0.41 ; kidney/blood partition coefficient

PRRH = 0.39 ; rapidly perfused tissue/blood partition coefficient (brain gut heart lung spleen)

PSRH = 0.34 ; slowly perfused tissue/blood partition coefficient (bone muscle skin)

;=============================================================

;Kinetic parameters

;=============================================================

; Absorption from GI-tract to liver

ka = 0.14 ; absorption rate constant {/hr} (calculated, details in manuscript)

Fa = 0.36 ; fraction of dose absorbed (calculated, details in manuscript)

;-------------------------------------------------------------------------------------------------------------------

; Excretion from liver via bile

kb = 1 ; excretion rate constant {/hr} for biliary excretion [31]

; Excretion to urine via glomerular filtration

GF = 1.8 ; (mL/min/kg bw), human glomerular filtration rate [32]

GFR = GF/1000*BW*60 ; (L/hr), scaled human glomerular filtration rate

;-------------------------------------------------------------------------------------------------------------------

;Metabolism of aloe-emodin in liver

; MPL: human liver microsomal protein yield scaling factor

MPL = 40 ; mg microsomal protein/g liver (QIVIVE tools [27])

; VLS9: human liver S9 protein yield scaling factor

VLS9 = 120.7 ; mg S9 protein/g liver [35]

;Maximum rate for metabolism of aloe-emodin to rhein, measured in vitro in the present study

VmaxRHc = 0.07859 {nmol/min/mg microsomal protein}

;Scaled maximum rate in liver

VmaxRH = VmaxRHc/1000*60*MPL*VL*1000 {µmol/hr}

;Michaelis-Menten constant for metabolism of aloe-emodin to rhein, measured in vitro in the present study

KmRH = 16.88 {uM}

; Maximum rate for metabolism of aloe-emodin to aloe-emodin glucuronide 1 (AG1), aloe-emodin glucuronide 2 (AG2), and aloe-emodin glucuronide 3 (AG3), measured in vitro in the present study

VmaxAG1c = 0.1291 {nmol/min/mg S9 protein}

VmaxAG2c = 0.1307 {nmol/min/mg S9 protein}

VmaxAG3c = 0.09611 {nmol/min/mg S9 protein}

;Scaled maximum rate in liver

VmaxAG1 = VmaxAG1c/1000*60*VLS9*VL*1000 {µmol/hr}

VmaxAG2 = VmaxAG2c/1000*60*VLS9*VL*1000 {µmol/hr}

VmaxAG3 = VmaxAG3c/1000*60*VLS9*VL*1000 {µmol/hr}

;Michaelis-Menten constant for metabolism of aloe-emodin to aloe-emodin glucuronide 1 (AG1), aloe-emodin glucuronide 2 (AG2), and aloe-emodin glucuronide 3 (AG3), measured in vitro in the present study

KmAG1 = 11.37 {µM}

KmAG2 = 9.776 {µM}

KmAG3 = 11.39 {µM}

;==============================================================

;Run settings

;==============================================================

;Molecular weight

MWAE = 270.24 ; Molecular weight (g/mol) of aloe-emodin

MWRH = 284.22 ; Molecular weight (g/mol) of rhein

;Oral dose

ODOSEmg = 40 ; given oral dose in mg/kg bw

ODOSEumol2 = ODOSEmg*1E-3/MWAE*1E6 ; recalculated to umol/kg bw

ODOSEumol = ODOSEumol2*BW ; given oral in umol

```
;time

Starttime = 0                    ; in hr

Stoptime = 24                    ; in hr


;=============================================================

;Main model calculations: aloe-emodin (AE)

;=============================================================

;GI compartment

;AStAE  = amount of aloe-emodin in GI tract, µmol

AStAE'  = -ka*AStAE

Init AStAE = ODOSEumol*Fa

;-------------------------------------------------------------------------------------------------------------------

;liver compartment

;ALAE = Amount of aloe-emodin in liver tissue, µmol

ALAE' = ka*AStAE + QL*(CBAE - CVLAE) - AbileAE'- AMLRH'-AMLG1'-AMLG2'-AMLG3'

Init ALAE = 0

CLAE = ALAE/VL

CVLAE = CLAE/PLAE


; AbileAE = amount of aloe-emodin excreted from liver via bile, µmol

AbileAE' = kb*ALAE

Init AbileAE = 0
```

;AMLRH = amount of aloe-emodin metabolized to rhein in liver, µmol

AMLRH' = (VmaxRH*CVLAE)/(KmRH + CVLAE)

Init AMLRH = 0

;AMLG1 = amount of aloe-emodin metabolized to aloe-emodin glucuronide 1 (AG1) in liver, µmol

AMLG1' = (VmaxAG1*CVLAE)/(KmAG1 + CVLAE)

Init AMLG1 = 0

;AMLG2 = amount of aloe-emodin metabolized to aloe-emodin glucuronide 2 (AG2) in liver, µmol

AMLG2' = (VmaxAG2*CVLAE)/(KmAG2 + CVLAE)

Init AMLG2 = 0

;AMLG3 = amount of aloe-emodin metabolized to aloe-emodin glucuronide 3 (AG3) in liver, µmol

AMLG3' = (VmaxAG3*CVLAE)/(KmAG3 + CVLAE)

Init AMLG3 = 0

;------------------------------------------------------------------------------------------------------------------

; kidney compartment

;AKAE = Amount of aloe-emodin in kidney tissue, umol

AKAE' = QK*(CBAE-CVKAE) -GFAE'

Init AKAE = 0

CKAE = AKAE/VK

CVKAE = CKAE/PKAE

;GFAE = amount of aloe-emodin excreted to urine via glomerular filtration, µmol

GFAE' = GFR*CVKAE

Init GFAE = 0

;--------------------------------------------------------------------------------------------------------------------

;fat compartment

;AFAE = amount of aloe-emodin in fat tissue, µmol

AFAE' = QF*(CBAE-CVFAE)

Init AFAE = 0

CFAE = AFAE/VF

CVFAE = CFAE/PFAE

;--------------------------------------------------------------------------------------------------------------------

;tissue compartment (rapidly perfused tissue)

;ARAE = amount of aloe-emodin  in rapidly perfused tissue, µmol

ARAE' = QR*(CBAE-CVRAE)

Init ARAE = 0

CRAE = ARAE/VR

CVRAE = CRAE/PRAE

;--------------------------------------------------------------------------------------------------------------------

;tissue compartment (slowly perfused tissue)

;ASAE = amount of aloe-emodin in slowly perfused tissue, µmol

ASAE' = QS*(CBAE-CVSAE)

Init ASAE = 0

CSAE = ASAE/VS

CVSAE = CSAE/PSAE

;--------------------------------------------------------------------------------------------------------------------

; blood compartment

;ABAE = amount of aloe-emodin in blood, µmol

ABAE' = QF*CVFAE + QL*CVLAE + QK*CVKAE + QS*CVSAE + QR*CVRAE -

(QF+QL+QK+QS+QR)*CBAE

Init ABAE = 0

CBAE = ABAE/VB

CPAE = CBAE/BPRAE ; plasma AE concentration

;==============================================================

;sub-model calculations: rhein (RH)

;==============================================================; ; liver compartment

; ALRH = amount of rhein in liver tissue, µmol

ALRH' = QL* (CBRH-CVLRH) + AMLRH'

Init ALRH = 0

CLRH = ALRH/VL

CVLRH = CLRH/PLRH

;--------------------------------------------------------------------------------------------------------------

; kidney compartment

;AKRH = amount of rhein in kidney tissue, umol

AKRH' = QK*(CBRH-CVKRH) -GFRH'

Init AKRH = 0

CKRH = AKRH/VK

CVKRH = CKRH/PKRH

;GFRH = amount of rhein excreted to urine via glomerular filtration, umol

GFRH' = GFR*CVKRH

Init GFRH = 0

;------------------------------------------------------------------------------------------------------------------

;fat compartment

;AFRH = amount of rhein in fat tissue, µmol

AFRH' = QF*(CBRH-CVFRH)

Init AFRH = 0

CFRH = AFRH/VF

CVFRH = CFRH/PFRH

;------------------------------------------------------------------------------------------------------------------

;tissue compartment (rapidly perfused tissue)

;ARRH = amount of rhein in rapidly perfused tissue, µmol

ARRH' = QR*(CBRH-CVRRH)

Init ARRH = 0

CRRH = ARRH/VR

CVRRH = CRRH/PRRH

;------------------------------------------------------------------------------------------------------------------

;tissue compartment (slowly perfused tissue)

;ASRH = amount of rhein in slowly perfused tissue, µmol

ASRH' = QS*(CBRH-CVSRH)

Init ASRH = 0

CSRH = ASRH/VS

CVSRH = CSRH/PSRH

```
;-----------------------------------------------------------------------------------------------------------------

; blood compartment

;ABRH = amount of rhein in blood, µmol

ABRH' = QF*CVFRH + QL*CVLRH + QK*CVKRH + QS*CVSRH + QR*CVRRH -
(QF+QL+QK+QS+QR)*CBRH

Init ABRH = 0

CBRH = ABRH/VB

CPRH = CBRH/BPRRH                  ; plasma RH concentration


;=================================================================

;Mass balance calculations of aloe-emodin

;=================================================================

TotalAE = ODOSEumol*Fa

CalculatedAE = AStAE + ALAE + AbileAE + AMLRH + AMLG1 + AMLG2 + AMLG3 + AKAE + GFAE + AFAE
+ ASAE + ARAE + ABAE


ERRORAE=(TotalAE-CalculatedAE)/(TotalAE+1E-30)*100

MASSBBALAE=TotalAE-CalculatedAE + 1


;=================================================================

;Mass balance calculations of rhein

;=================================================================

TotalRH = AMLRH

CalculatedRH = ALRH + AKRH + GFRH + AFRH + ASRH + ARRH + ABRH
```

ERRORRH=(TotalRH-CalculatedRH)/(TotalRH+1E-30)*100

MASSBBALRH=TotalRH-CalculatedRH + 1

**References**


(1) Liu, Y.; Mapa, M. S. T.; Sprando, R. L. Liver Toxicity of Anthraquinones: A Combined in Vitro Cytotoxicity and in Silico Reverse Dosimetry Evaluation. *Food Chem. Toxicol.* **2020**, *140*, 111313. https://doi.org/10.1016/j.fct.2020.111313.

(2) Chiu, W. A.; Barton, H. A.; DeWoskin, R. S.; Schlosser, P.; Thompson, C. M.; Sonawane, B.; Lipscomb, J. C.; Krishnan, K. Evaluation of Physiologically Based Pharmacokinetic Models for Use in Risk Assessment. *Journal of Applied Toxicology: An International Journal* **2007**, *27* (3), 218–237.

(3) Rietjens, I. M.; Louisse, J.; Punt, A. Tutorial on Physiologically Based Kinetic Modeling in Molecular Nutrition and Food Research. *Molecular nutrition & food research* **2011**, *55* (6), 941–956.

(4) Shi, F.; Chen, L.; Wang, Y.; Liu, J.; Adu-Frimpong, M.; Ji, H.; Toreniyazov, E.; Wang, Q.; Yu, J.; Xu, X. Enhancement of Oral Bioavailability and Anti-Hyperuricemic Activity of Aloe Emodin via Novel Soluplus®—Glycyrrhizic Acid Mixed Micelle System. *Drug Deliv. and Transl. Res.* **2022**, *12* (3), 603–614. https://doi.org/10.1007/s13346-021-00969-8.

(5) Yu, C.-P.; Shia, C.-S.; Lin, H.-J.; Hsieh, Y.-W.; Lin, S.-P.; Hou, Y.-C. Analysis of the Pharmacokinetics and Metabolism of Aloe-Emodin Following Intravenous and Oral Administrations in Rats: Pharmacokinetics and Metabolism of Aloe-Emodin. *Biomed. Chromatogr.* **2016**, *30* (10), 1641–1647. https://doi.org/10.1002/bmc.3735.

(6) Dong, X.; Fu, J.; Yin, X.; Yang, C.; Ni, J. Aloe-emodin Induces Apoptosis in Human Liver HL-7702 Cells through Fas Death Pathway and the Mitochondrial Pathway by Generating Reactive Oxygen Species. *Phytotherapy Research* **2017**, *31* (6), 927–936.

(7) Dong, X.; Fu, J.; Yin, X.; Qu, C.; Yang, C.; He, H.; Ni, J. Induction of Apoptosis in HepaRG Cell Line by Aloe-Emodin through Generation of Reactive Oxygen Species and the Mitochondrial Pathway. *Cell. Physiol. Biochem.* **2017**, *42* (2), 685–696. https://doi.org/10.1159/000477886.

(8) Liu, D. ming; Yang, D.; Zhou, C. yan; Wu, J. si; Zhang, G. lin; Wang, P.; Wang, F.; Meng, X. Aloe-Emodin Induces Hepatotoxicity by the Inhibition of Multidrug Resistance Protein 2. *Phytomedicine* **2020**, *68*, 153148.

(9) Lu, G. D.; Shen, H.-M.; Ong, C. N.; Chung, M. C. Anticancer Effects of Aloe-emodin on HepG2 Cells: Cellular and Proteomic Studies. *PROTEOMICS–Clinical Applications* **2007**, *1* (4), 410–419.

(10) Zhu, S.; Jin, J.; Wang, Y.; Ouyang, Z.; Xi, C.; Li, J.; Qiu, Y.; Wan, J.; Huang, M.; Huang, Z. The Endoplasmic Reticulum Stress Response Is Involved in Apoptosis Induced by Aloe-Emodin in HK-2 Cells. *Food Chem. Toxicol.* **2012**, *50* (3–4), 1149–1158. https://doi.org/10.1016/j.fct.2011.12.018.

(11) Hu, Y.; Quan, Z.; Li, D.; Wang, C.; Sun, Z. Inhibition of CYP3A4 Enhances Aloe-Emodin Induced Hepatocyte Injury. *Toxicol. In Vitro* **2022**, *79*, 105276. https://doi.org/10.1016/j.tiv.2021.105276.

(12) Brown, R. P.; Delp, M. D.; Lindstedt, S. L.; Rhomberg, L. R.; Beliles, R. P. Physiological Parameter Values for Physiologically Based Pharmacokinetic Models. *Toxicol Ind Health* **1997**, *13* (4), 407–484. https://doi.org/10.1177/074823379701300401.

(13) Gearhart, J. M.; Jepson, G. W.; Clewell III, H. J.; Andersen, M. E.; Conolly, R. B. Physiologically Based Pharmacokinetic and Pharmacodynamic Model for the Inhibition of Acetylcholinesterase by Diisopropyfluorophosphate. *Toxicology and applied pharmacology* **1990**, *106* (2), 295–310.

(14) Kumar, S.; Yadav, M.; Yadav, A.; Rohilla, P.; Yadav, J. P. Antiplasmodial Potential and Quantification of Aloin and Aloe-Emodin in Aloe Vera Collected from Different Climatic Regions of India. *BMC complementary and alternative medicine* **2017**, *17*, 1–10.

(15) Zhu, W.; Wang, X.-M.; Zhang, L.; Li, X.-Y.; Wang, B.-X. Pharmacokinetic of Rhein in Healthy Male Volunteers Following Oral and Retention Enema Administration of Rhubarb Extract: A Single Dose Study. *The American Journal of Chinese Medicine* **2005**, *33* (06), 839–850.

(16) Sun, S.-W.; Yeh, P.-C. Analysis of Rhubarb Anthraquinones and Bianthrones by Microemulsion Electrokinetic Chromatography. *Journal of pharmaceutical and biomedical analysis* **2005**, *36* (5), 995–1001.

(17) Wang, J.; Li, H.; Jin, C.; Qu, Y.; Xiao, X. Development and Validation of a UPLC Method for Quality Control of Rhubarb-Based Medicine: Fast Simultaneous Determination of Five Anthraquinone Derivatives. *Journal of pharmaceutical and biomedical analysis* **2008**, *47* (4–5), 765–770.

(18) Wang, Z.-W.; Wang, J.-S.; Yang, M.-H.; Luo, J.-G.; Kong, L.-Y. Developmental Changes in the Composition of Five Anthraquinones from Rheum Palmatum as Quantified by 1H-NMR. *Phytochemical Analysis* **2013**, *24* (4), 329–335.

(19) Duke, J. A. *Database of Phytochemical Constituents of GRAS Herbs and Other Economic Plants*; CRC Press, 1992.

(20) Wang, J.; Zhao, H.; Zhao, Y.; Jin, C.; Liu, D.; Kong, W.; Fang, F.; Zhang, L.; Wang, H.; Xiao, X. Hepatotoxicity or Hepatoprotection? Pattern Recognition for the Paradoxical Effect of the Chinese Herb Rheum Palmatum L. in Treating Rat Liver Injury. *PLoS One* **2011**, *6* (9), e24498.

(21) Wu, W.; Yan, R.; Yao, M.; Zhan, Y.; Wang, Y. Pharmacokinetics of Anthraquinones in Rat Plasma after Oral Administration of a Rhubarb Extract: PK of Rhubarb Extract in Rats. *Biomed. Chromatogr.* **2014**, *28* (4), 564–572. https://doi.org/10.1002/bmc.3070.

(22) Liu, Y.-H.; Huang, Z.-H.; Dong, L.; Pei, W.-X.; Sun, Y.; Gao, X.-Y. Simultaneous Content Determination of 14 Components in Rhei Radix et Rhizoma by High Performance Liquid Chromatography Method. *Zhongguo*

*Zhong yao za zhi= Zhongguo Zhongyao Zazhi= China Journal of Chinese Materia Medica* **2017**, *42* (23), 4514–4519.

(23) Fang, F.; Wang, J.; Zhao, Y.; Jin, C.; Kong, W.; Zhao, H.; Wang, H.; Xiao, X. A Comparative Study on the Tissue Distributions of Rhubarb Anthraquinones in Normal and CCl4-Injured Rats Orally Administered Rhubarb Extract. *Journal of ethnopharmacology* **2011**, *137* (3), 1492–1497.

(24) Xu, L.; Chan, C.; Lau, C.; Yu, Z.; Mok, D. K. W.; Chen, S. Simultaneous Determination of Eight Anthraquinones in Semen Cassiae by HPLC-DAD: Simultaneous Determination Anthraquinones in Semen Cassiae. *Phytochem. Anal.* **2012**, *23* (2), 110–116. https://doi.org/10.1002/pca.1331.

(25) Jiang, T.-F.; Lv, Z.-H.; Wang, Y.-H. Separation and Determination of Anthraquinones inCassia Obtusifolia (Leguminosae) by Micellar Electrokinetic Capillary Electrophoresis. *J. Sep. Science* **2005**, *28* (16), 2225–2229. https://doi.org/10.1002/jssc.200500144.

(26) Ren, Q.; Bakker, W.; de Haan, L.; Rietjens, I. M.; Bouwmeester, H. Induction of Nrf2-EpRE-Mediated Gene Expression by Hydroxyanthraquinones Present in Extracts from Traditional Chinese Medicine and Herbs. *Food Chem. Toxicol.* **2023**, *176*, 113802. https://doi.org/10.1016/j.fct.2023.113802.

(27) Punt, A.; Pinckaers, N.; Peijnenburg, A.; Louisse, J. Development of a Web-Based Toolbox to Support Quantitative In-Vitro-to-In-Vivo Extrapolations (QIVIVE) within Nonanimal Testing Strategies. *Chem. Res. Toxicol.* **2021**, *34* (2), 460–472. https://doi.org/10.1021/acs.chemrestox.0c00307.

(28) Rodgers, T.; Rowland, M. Physiologically based pharmacokinetic modelling 2: predicting the tissue distribution of acids, very weak bases, neutrals and zwitterions. *Journal of pharmaceutical sciences* **2006**, *95* (6), 1238–1257. https://doi.org/10.1002/jps.20502.

(29) Hallifax, D.; Foster, J. A.; Houston, J. B. Prediction of Human Metabolic Clearance from in Vitro Systems: Retrospective Analysis and Prospective View. *Pharmaceutical research* **2010**, *27*, 2150–2161.

(30) Hao, K.; Qi, Q.; Wan, P.; Zhang, J.; Hao, H.; Liang, Y.; Xie, L.; Wang, G.; Sun, J. Prediction of Human Pharmacokinetics from Preclinical Information of Rhein, an Antidiabetic Nephropathy Drug, Using a Physiologically Based Pharmacokinetic Model. *Basic Clin. Pharmacol. Toxicol.* **2014**, *114* (2), 160–167. https://doi.org/10.1111/bcpt.12148.

(31) Wang, D.; Rietdijk, M. H.; Kamelia, L.; Boogaard, P. J.; Rietjens, I. M. C. M. Predicting the in Vivo Developmental Toxicity of Benzo[a]Pyrene (BaP) in Rats by an in Vitro–in Silico Approach. *Arch. Toxicol.* **2021**, *95* (10), 3323–3340. https://doi.org/10.1007/s00204-021-03128-7.

(32) Walton, K.; Dorne, J. L. C. M.; Renwick, A. G. Species-Specific Uncertainty Factors for Compounds Eliminated Principally by Renal Excretion in Humans. *Food Chem. Toxicol.* **2004**, *42* (2), 261–274. https://doi.org/10.1016/j.fct.2003.09.001.

(33) Zhang, D.; Luo, G.; Ding, X.; Lu, C. Preclinical Experimental Models of Drug Metabolism and Disposition in Drug Discovery and Development. *Acta Pharm. Sin. B* **2012**, *2* (6), 549–561. https://doi.org/10.1016/j.apsb.2012.10.004.

(34) Houston, J. B. Utility of in Vitro Drug Metabolism Data in Predicting in Vivo Metabolic Clearance. *Biochem. Pharmacol.* **1994**, *47* (9), 1469–1479. https://doi.org/10.1016/0006-2952(94)90520-7.

(35) Cubitt, H. E.; Houston, J. B.; Galetin, A. Prediction of Human Drug Clearance by Multiple Metabolic Pathways: Integration of Hepatic and Intestinal Microsomal and Cytosolic Data. *Drug Metab. Dispos.* **2011**, *39* (5), 864–873. https://doi.org/10.1124/dmd.110.036566.
